# Investigating the effects of chronic low-dose radiation exposure in the liver of a hypothermic zebrafish model

**DOI:** 10.1038/s41598-022-26976-4

**Published:** 2023-01-17

**Authors:** Thomas Cahill, Willian Abraham da Silveira, Ludivine Renaud, Hao Wang, Tucker Williamson, Dongjun Chung, Sherine Chan, Ian Overton, Gary Hardiman

**Affiliations:** 1 School of Biological Sciences and Institute for Global Food Security, Queens University Belfast, Belfast, BT9 5DL UK; 2 School of Health, Science and Wellbeing, Department of Biological Sciences, Science Centre, Staffordshire University, Leek Road, Stoke-On-Trent, ST4 2DF UK; 3 International Space University, 1 Rue Jean-Dominique Cassini, 67400 Illkirch-Graffenstaden, France; 4 Department of Medicine, Medical University of South Carolina, Charleston, SC 29425 USA; 5 Department of Drug Discovery and Biomedical Sciences, Medical University of South Carolina, Charleston, SC 29425 USA; 6 Department of Biomedical Informatics, The Ohio State University, Columbus, OH 43210 USA; 7 JLABS at the Children’s National Research and Innovation Campus, Washington, DC 20012 USA; 8 Patrick G Johnston Centre for Cancer Research, Queen’s University Belfast, Belfast, BT9 7AE UK

**Keywords:** Biochemistry, RNA, Regulatory networks, Systems analysis

## Abstract

Mankind’s quest for a manned mission to Mars is placing increased emphasis on the development of innovative radio-protective countermeasures for long-term space travel. Hibernation confers radio-protective effects in hibernating animals, and this has led to the investigation of synthetic torpor to mitigate the deleterious effects of chronic low-dose-rate radiation exposure. Here we describe an induced torpor model we developed using the zebrafish. We explored the effects of radiation exposure on this model with a focus on the liver. Transcriptomic and behavioural analyses were performed. Radiation exposure resulted in transcriptomic perturbations in lipid metabolism and absorption, wound healing, immune response, and fibrogenic pathways. Induced torpor reduced metabolism and increased pro-survival, anti-apoptotic, and DNA repair pathways. Coupled with radiation exposure, induced torpor led to a stress response but also revealed maintenance of DNA repair mechanisms, pro-survival and anti-apoptotic signals. To further characterise our model of induced torpor, the zebrafish model was compared with hepatic transcriptomic data from hibernating grizzly bears (*Ursus arctos horribilis*) and active controls revealing conserved responses in gene expression associated with anti-apoptotic processes, DNA damage repair, cell survival, proliferation, and antioxidant response. Similarly, the radiation group was compared with space-flown mice revealing shared changes in lipid metabolism.

## Introduction

As we enter a new era of space exploration that will be defined by manned interplanetary travel, as detailed in the Artemis programme^1^, we must develop innovative countermeasures against the adverse effects of microgravity and radiation^2–4^. The concept of using synthetic torpor is gaining attention given the observation that the use of torpor in nature provides resistance to bone and muscle atrophy during disuse, as seen in the yellow-bellied marmot and the hibernating brown bear. This suggests a role for torpor in preventing these adverse outcomes in the microgravity environment^5,6^. However, little is known about the genetic mechanisms that regulate such specific adaptations, and replicating these may prove challenging. This study focuses on replicating one of the most fundamental and conserved aspects of torpor: hypometabolism, considered to be the main contributor to radioprotection. There already exists compelling evidence of torpor conferring radio-protective effects^7,8^, highlighted in experiments involving Balb/c mice undergoing therapeutic hypothermia following a large dose of radiation that led to increased survival times, compared with controls^9^. Similarly, the use of AMP to induce a hypometabolic state in mice was found to decrease radio-sensitivity^10^. However, additional work is needed to define the molecular mechanisms behind this conferred radioprotection.

Natural torpor describes a change in physiology characterised by a hypometabolic state normally facilitated by a coordinated reduction in body temperature, heart rate, locomotion, respiration, and brain activity^11,12^. Heterothermic animals that utilise torpor tend to do so in either a daily fashion where torpor bouts last less than 24 h (daily torpor) or in consecutive or continuous bouts, often referred to as hibernation^13^. Torpor is often considered a stress response, as the reduction in metabolic rate is exploited to conserve energy during cold periods or food scarcity and its use has been found to increase survival rates during harsh times^14^. Furthermore, it is thought that a reduction in respiration and perfusion along with a decrease in hemoglobin oxygen unloading during hibernation results in reduced cellular oxygen supply, as hypoxia-related genes are upregulated during hibernation^15^. It has been suggested that this reduction in oxygen concentration provides radio-protective effects by limiting the generation of ROS in what’s known as the ‘oxygen effect’, in the same way that hypoxia confers radio-resistance to tumours^16^. There may be value in exploiting ectotherms in further characterising this response as zebrafish (*Danio rerio*) body temperature, and thus metabolic rate can be easily managed through changes in ambient temperatures. Additionally, zebrafish acclimated at 18 °C have shown reduced oxygen consumption^17^, and hypoxia-related genes have also been found to be upregulated in other ectothermic animals such as the crucian carp during cold acclimatisation in both hypoxic and normoxic conditions^18^. Furthermore, zebrafish offer an attractive low-cost experimental model due to their ease of maintenance, rapid development, genetic similarity to humans (~ 70%), and the availability of a high-quality reference genome^19,20^.

The liver plays a central role in carbohydrate and lipid metabolism, detoxification, immunity, and bile formation, and preserving liver health will play a vital role in maintaining astronaut health during long-term space travel^21^. However, spaceflight results in physical and pathological changes to the liver as evidenced in rodent studies exposed to short-duration flight (13 days) revealing morphological changes in the liver involving a reduction in mass^22^. Others report a decrease in Kupffer cells^23^, diminished levels of cytochrome P-450^24^ glutathione^25^, and cholesterol^26^. Studies have also shown that the liver of mice exposed to both radiation or spaceflight have increased pro-fibrogenic markers involving genes that regulate stellate cell activation and epithelial-to-mesenchymal transition (EMT)^27,28^. Moreover, the discovery that lipid droplets accumulate in mice's liver after spaceflight points to spaceflight as a risk factor for non-alcoholic fatty liver disease (NAFLD) (3). Data from mice livers exposed to low-dose radiation (30–120 mGy) and collected 60 weeks after irradiation also suggest that dysfunction in lipid metabolism is a long-term effect^29^. Similarly, changes in insulin sensitivity following spaceflight results in sub-clinical diabetic phenotypes^30^ and analysis of hepatic transcriptomes in space-flown mice identified increased expression of genes involved in the oxidative stress response^31^, fatty acid and glucose metabolism, ribosome function, protein folding, and the adaptive immune response^32^.

We wanted to explore transcriptomic events that may regulate the radioprotective effects of a hypothermic/hypometabolic state in the liver. Therefore, we exploited the ectothermic nature of zebrafish to establish various experimental and control groups with reduced body temperatures/metabolism and radiation exposure (0.3 Gy) to simulate the amount of radiation that would be experienced on a journey to Mars. Melatonin was used as a sedative, which has been shown to promote a sleep-like state in zebrafish by reducing locomotor activity and elevating arousal threshold^33^. In addition, it is a potent antioxidant, shown to have radioprotective effects^34^ and its use has been recorded to improve the severity of NAFLD^35^ and diabetes^36^. In this study, we utilized melatonin to decrease locomotion and investigated its therapeutic potential against the effects of radiation exposure. We previously used this hypometabolism + melatonin model (induced torpor) to demonstrate a reduction in activity and the expression of metabolic genes in the gastrointestinal tract (GIT). We also observed an increase in protein maintenance genes, as well as in anti-apoptotic, proliferative, and pro-survival signalling. On the other hand, our results showed that exposure to low-dose radiation caused DNA damage, oxidative stress, glucocorticoid signalling, and cell cycle arrest. When irradiated under induced/synthetic torpor, radiation-induced stress signals were observed, however, the increase in anti-apoptotic, mitogenic, and pro-survival signals were all maintained, suggesting radio-protective effects^37^. Here we report our findings in the context of hepatic health. Our results show that low-dose radiation leads to changes in genes involved in cell death, lipid metabolism and transport, the immune system, and the extracellular matrix. Additionally, like that observed in the GIT, the results from the induced torpor group show an increase in gene expression of pro-survival, proliferative, anti-apoptotic, and DNA repair genes. We explored the role of melatonin as a potential therapeutic for the lipid dysregulation that occurs after exposure to low dose radiation and performed a cross-species comparison of the zebrafish groups with space flown mice and hibernating bear to characterise the utility of the zebrafish models.

## Results

### Induced Torpor in zebrafish activates DNA repair and mitogenic pathways

**Figure 1:**
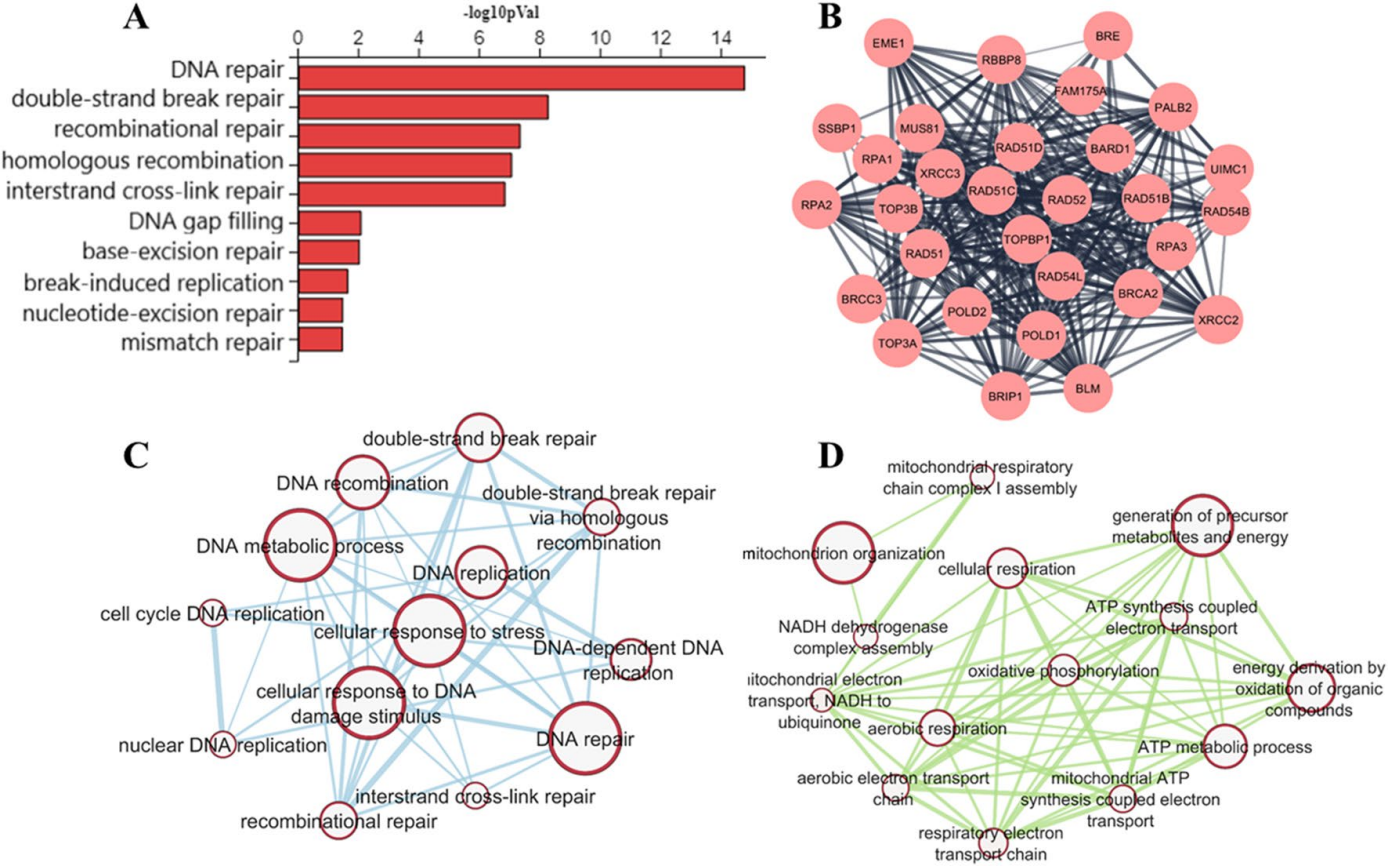
(**A**) Enriched biological processes related to DNA repair pathways including homologous recombination (GO:0000725), base excision repair (GO:0006284), nucleotide excision repair (GO:0006289) and mismatch repair (GO:0006298). (**B**) A protein interaction network generated using STRING of genes differentially expressed in the Homologous recombination pathway (KEGG: 03440)^39^. (**C**) Enrichment map cluster of upregulated gene sets relating to DNA repair processes. (**D**)Enrichment map cluster of downregulated metabolic processes.

To characterise the effects of an induced-torpor-like state on the zebrafish liver, differential expression analysis (DEA) was carried out on the induced-torpor group vs control (18.5-mel vs 28.5-Ctrl). This revealed 1986 upregulated and 765 downregulated genes (FC ± 1.5, *q* ≤ 0.1) (Supplementary Table S4) that were then subject to over-representation (ORA) and impact analyses performed (Supplementary Tables S5, S6). Analysis of upregulated genes revealed cell cycle-related processes (GO:0051301, GO:0000280) involving cyclins (*CycA, CycE)* and cyclin-dependent kinases (*CDK1),* along with chromosome maintenance genes (*Mcm2, Mcm4, and Mcm5*). Additionally, while the zebrafish were acclimatised to reduced temperatures, it is understood that cold stress can result in oxidative stress that can damage DNA^38^. Here, we observed increased DNA damage response processes including p53 signalling (KEGG: 04115)^39^, comprised of genes involved in DNA damage sensing (*ATR),* mitotic checkpoint (*Chek1* and *Chek2),* and a cell cycle arrest (*CDKN1C)*. Hence, multiple DNA repair pathways were also upregulated (shown in Fig. 1A,C) including, DNA mismatch repair (KEGG: 03430)^39^ (Figure S2), nucleotide excision repair (KEGG: 03420)^39^ (Figures S3), base excision repair (KEGG: 03410)^39^ (Figure S4) and the homologous recombination pathways (KEGG: 03440)^39^ presented in Figure S5, showing upregulation of genes involved in recognition of double-strand breaks, strand invasion, displacement, and ligation. A protein interaction network containing these genes is presented in Fig. 1B. We also note the upregulation of a sirtuin family member (*SIRT6)* which stimulates homologous/non-homologous repair at double-strand breaks^40^.

It has previously been established that energy reserves, measured by a hepatosomatic index, were found to increase in zebrafish acclimated at 18° C, suggesting decreased energy use at lower temperatures^17^. Consistently, ORA of downregulated genes revealed several metabolic processes (Fig. 1D, Supplementary Table S5) including oxidative phosphorylation (PW:0000034), electron transport chain (ETC) (M39417), glycolysis, gluconeogenesis (M39474), and lipid metabolism including cholesterol metabolism (M39853), Leptin signalling (M39491), and Peroxisomes (M6391). The FOXA2 and FOX3 transcription factor network gene set was also significantly perturbed. *FOXA2* regulates aerobic glycolysis^41^, in line with increased hepatic carbohydrate concentrations found at lower temperatures^17^ while *FOXA3* is important for glucose metabolism and lipid biosynthesis^42^. Similarly, the ribosome pathway (M189) and translation GO term (GO:0006412) were downregulated suggesting the conservation of biochemical energy^43^. Furthermore, immune-related TNF and IL-1 signalling pathways were enriched consistent with suppression of innate and adaptive immunity as seen in hibernating animals^44,45^, as well as JNK activated MAPK pathway which regulates the response to various stressors^46^.

To explore tissue-specific responses to the induced torpor-like state we performed a cross-comparison of differentially expressed genes (DEGs) in the liver with those previously reported in the GIT^37^. ORA of the shared upregulated genes (378, FC + 1.5, *q* 0.1) (Supplementary Table S7) revealed enrichment of the cell cycle, spliceosome, DNA damage response and DNA repair pathways including DNA mismatch repair and nucleotide excision repair. Also upregulated was the *FOXM1* transcription factor network pathway, which is induced in a ROS or hypoxia-dependent manner to initiate proliferation, suppress apoptosis, and promote survival in response to stressors^47,48^. Given these links to hypoxia, we investigated hypoxia-related genes in both tissues. We found upregulation of *hif1al* (*q* = 0.006) in the liver and the upregulation of *hif1aa* in both the liver and GIT (*q* = 0.08 & 0.02, respectively). We also observed a significant downregulation of the *PHD3* gene (*EGLN3,* FC −1.89, *q* = 0.01) involved in HIF hydroxylation and degradation. ORA of the shared downregulated genes (261, FC −1.5, *q* = 0.1) (Supplementary Table S7) revealed shared downregulation of metabolic processes including glycolysis, gluconeogenesis, pyruvate metabolism, tricarboxylic acid cycle (TCA) cycle, peroxisome pathway, and the ETC, which is central to oxidative phosphorylation.

### Low dose radiation causes a robust change in the expression of immune response genes in zebrafish liver

**Figure 2:**
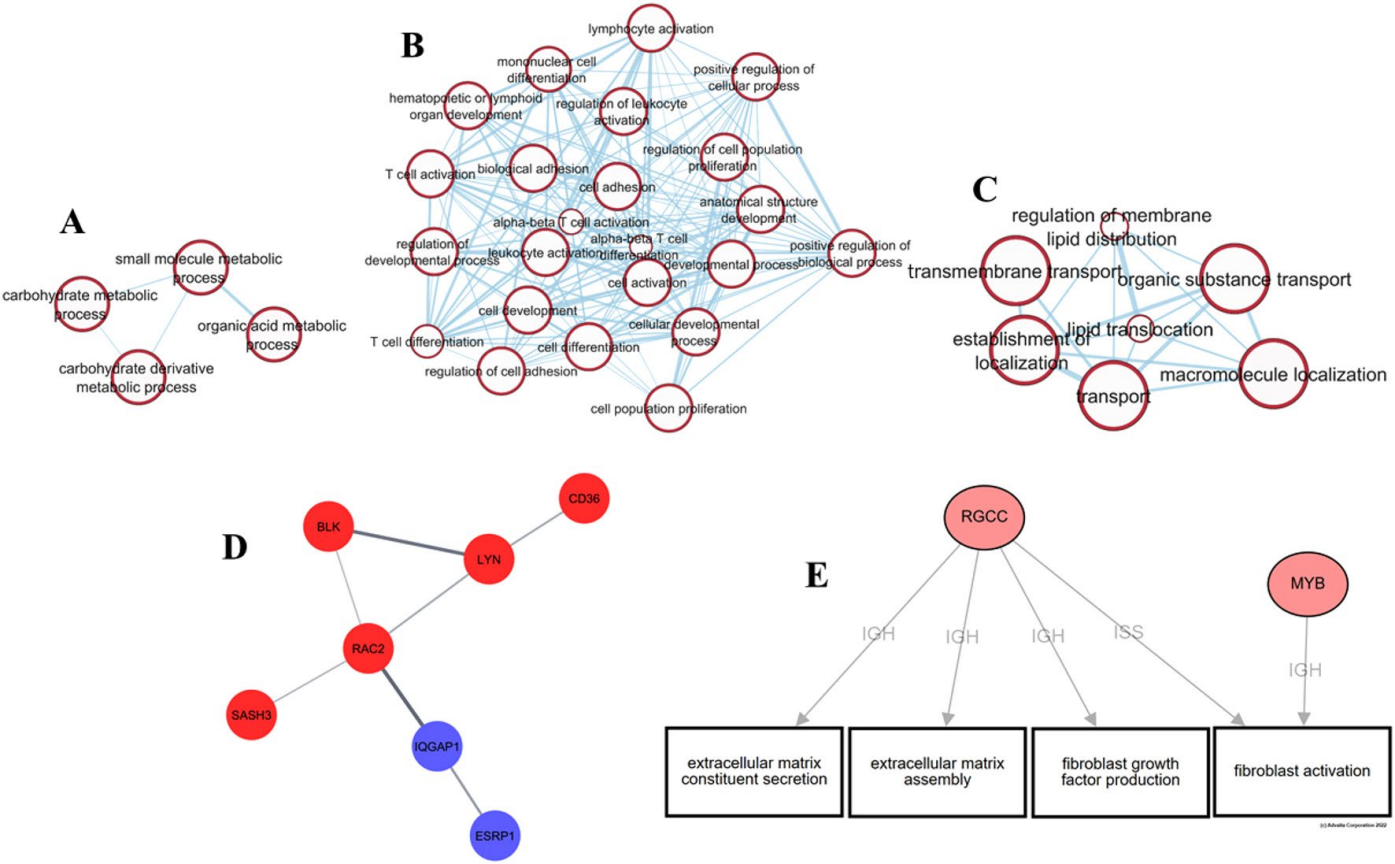
(**A**) Enrichment map of genes upregulated in response to radiation showing GO terms related to carbohydrate metabolism. (**B**) GO terms upregulated in response to radiation, showing enrichment of several immune-related processes such as lymphocyte, leucocyte, and T cell activation (generated using Enrichment map). (**C**)Enrichment map of genes upregulated in response to radiation showing changes in lipid transport. (**D**) Protein interaction map generated using STRING of genes from the GO term of immune effector process GO term (GO:0002699) (red) with genes from the response to fibroblast growth factor (GO:0071774) (blue). (**E**) iPathwayGuide network constructed with genes from the Immune processes GO term and BP terms related to ECM and fibroblast activation.

Exposure to low dose radiation (LDR) during inter-planetary travel is one of the major challenges to maintaining health during spaceflight. To determine the effects of 0.32 Gy of gamma radiation on the zebrafish liver we performed DEA on the radiation compared with the control group (28.5-rad vs 28.5-Ctrl) (Supplementary Table S8) and carried out ORA and impact analysis on the DEGs (Supplementary Table S9 & S10). Upregulated genes (542, FC + 1.5, *q* ≤ 0.1) revealed cell death-related pathways such as necroptosis (KEGG: 04217, KEGG: 04216, GO:0048102)^39^. This was accompanied by the wound healing response (GO:0009611 GO:0042060) (shown in Fig. 2), and involved the upregulation of a plasminogen activator, *Plau*. Lipid transport and metabolism were also perturbed (GO:0006869, GO:0016042, Fig. 2C), along with the PPAR signalling pathway involving *FABP2*, *CD36*, and *SLC27A4*, consistent with studies demonstrating spaceflight-induced lipid accumulation in the liver^32^. Similarly, the results suggest disturbed liver function with upregulation of *ABCC4,* which functions in bile acid secretion. Carbohydrate metabolism which is known to be upregulated in response to stress^49^ was also enriched in contrast to the induced torpor group (GO:0044262, GO:1901135, Fig. 2A), which involved upregulation of the glycolic enzyme gene *PFKFB3*. Immune-related GO terms were also upregulated including, immune cell activation (GO:0046649), differentiation (GO:0002292), proliferation (GO:0032943), and cell adhesion (GO:0007159) (shown in Fig. 2B). Furthermore, fibrogenic GO terms were upregulated such as fibroblast growth factor (GO:0071774) and fibroblast activation (GO:0072537). This was supplemented by the extracellular matrix (ECM) receptor interaction pathway involving the upregulation of collagen and laminin genes (*COL6A2, LAMB4*) that comprise the ECM, as well as cell adhesion with laminin and integrin genes (*ITGB4, LAMB4*), indicative of cell remodelling.

Liver fibrogenesis is mediated in an immune and inflammatory-dependent manner^50^ whereby TGF-β stimulates EMC production by initiating the epithelial-mesenchymal transition (EMT) of hepatocytes leading to the acquisition of mesenchymal properties capable of producing ECM proteins^51^. These results showed enrichment of TGF-β secretion (GO:0038044) and cytoskeleton organisation (GO:0051493, GO:0051125, GO:0110053), suggesting cell remodelling associated with epithelial-mesenchymal plasticity. Using STRING, we identified protein interactions between *RAC2,* from immune effector process GO term (GO:0002699) and *IQGAP1* from the response to fibroblast growth factor GO term (GO:0071774) as seen in Fig. 2D. *RAC2* is involved in cell polarity while *IQGAP2* interacts with cell adhesion molecules, and signalling molecules involved in cell morphology and motility. Additionally, network analysis with genes involved in the immune process and GO terms involved in ECM and fibroblast activation suggest that *RGCC* and *MYB* play roles in linking these processes. Downregulated GO terms (159, FC -1.5, *q* ≤ 0.1) included cell growth (GO:0016049), carbohydrate metabolism (GO:0005975), and cell and neuron morphogenesis (GO:0035239, GO:0048812, GO:0048858).

Again, a comparison was made between DEGs of the LDR groups of the liver (FC ± 1.5, *q* ≤ 0.1) and GIT (FC ± 1.5, *q* ≤ 0.4) to look for shared responses (Supplementary Table S11). Performing ORA on genes revealed shared upregulation of cell cycle checkpoints GO terms (PW:0000096, PW:0000385) as well as in lipid metabolism (GO:0006629), ABC and sodium-glucose transporters (M11911, PW:0000561), and the renin-angiotensin system (M17636).

### Induced torpor + radiation leads to a mixed phenotype

**Figure 3:**
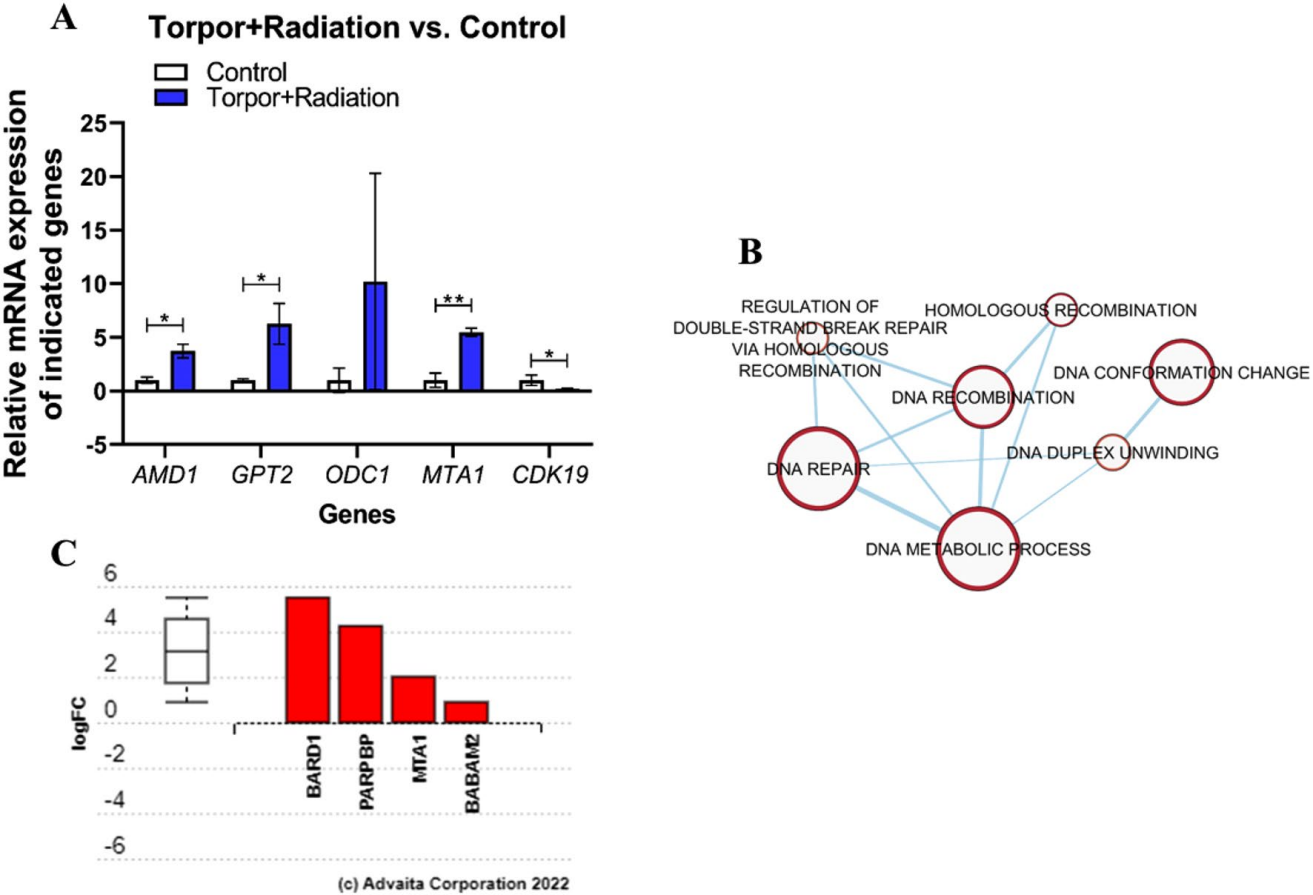
(**A**) Gene expression in the induced torpor + radiation (18.5-mel-rad) group compared to that in the control (28.5-Ctrl) group. The figure shows the relative increase in AMD1 (*p* = 0.012), *GTP1* (*p* = 0.034), *ODC1* (*p* = 0.309), and *MTA1* (*p* = 0.0022) expression compared to the control. *ODC1* shows a trend towards increased expression in the torpor + radiation group vs control (28.5-Ctrl). *CDK19* shows decreased expression compared to the control group (*p* = 0.05) *p ≤ 0.05 and ** ≤ 0.01. (**B**) Enrichment map showing that genes related to DNA repair-related biological processes such as homologous recombination are upregulated relative to the control group. (**C**) Bar plot showing differential expression of DNA repair process genes in induced torpor + radiation vs control. *BARD1* and *PARPBP* show strong upregulation in the induced torpor + radiation condition.

To determine whether an induced torpor-like state would be protective against LDR we exposed an induced torpor group to 0.32 Gy of gamma radiation and compared transcriptomic responses with the control group (18.5-mel-rad vs 28.5-Ctrl) (*q* ≤ 0.4) (Supplementary Table S12). The resulting 473 DEGs were subjected to ORA and impact analysis (Supplementary Table S13 & S14). Similar to the radiation group we observed upregulation of GO terms including a response to ionising radiation (GO:0010212), DNA damage checkpoints (GO:0000077), and cell cycle arrest (GO:0045930). This involved the upregulation of a CDK inhibitor (*CDKN1C)* and the downregulation of *CDK19*, which was validated with qPCR (*p* = 0.05) (Fig. 3). Changes to the circadian rhythm GO term (GO:0007623) involved the upregulation of *PER2, NR1D1,* and the downregulation of *PER3;* consistent with previous work relating radiation exposure to alterations in the circadian rhythm^52^. Developmental GO terms of the liver (GO:0001889) and hepatobiliary system (GO:0061008) were enriched as well as the positive regulation of bile acid biosynthetic and metabolic processes (GO:1904,253) indicating perturbed liver function. The results also indicate an increase in carbohydrate metabolism (GO:0044262), including upregulation of *GALK1* and enrichment of acetyl-CoA metabolism (GO:0006084)*.*

However, results much like that of the induced torpor-only group were present. We noted the enrichment of GO terms related to cell division (GO:0051301, GO:0000082, GO:0044770, GO:0051304), including Aurora B and Aurora A signalling pathways (M7963, M14, M242), which are important for chromosomal segregation^43^. Likewise, several DNA repair-related processes (GO:0006281) were upregulated (Fig. 3B) involving the expression of *BABAM2, MTA1,* and *PARPBP* genes (Fig. 3C). The qPCR analysis of the *MTA1* gene also showed a significant increase in the expression of *MTA1* (*p* = 0.0022) (Fig. 3A) in the torpor + radiation group compared to the control. Additionally, the hypoxia-related FOXM1 transcription factor network (M176) was enriched. Furthermore, the results also point to the biosynthesis and metabolism of polyamines such as spermidine and putrescine that involve upregulation of *AMD1* and *OCD1.* The quantification of *AMD1* by qPCR confirmed a significant increase in expression in the torpor + radiation group compared to the control group (*p* = 0.012, Fig. 3). Q-PCR of *ODC1* showed a trend towards an increase in expression, although the results were not significant (*p* = 0.309, Fig. 3). The ORA of the downregulated genes showed GO terms related to protein translation and folding in the endoplasmic reticulum (GO:0043038, GO:0034975), as well as mitochondrial membrane genes (GO:005741, GO:0031968).

A direct comparison of DEGs between the torpor + radiation and the radiation-only group (*q* ≤ 0.4) was made to determine unique and shared responses (Supplementary Table S15). It revealed shared upregulation of 195 genes, most of which played a role in the positive and negative regulation of the cell cycle (GO:0140014 GO:0045841), as well as polyamine metabolism and DNA repair (GO:0070531). Next, 85 downregulated DEGs were shared which were involved in protein synthesis (GO:0043038). Four genes were upregulated in the radiation group and downregulated in the torpor + radiation group which suggests increased innate immune signalling (*RSAD2*), an anti-inflammatory gene (*IKBKE*) the expression of which is stimulated by pro-inflammatory ligands such as TNFα, IL-1β, IFN-γ and LPS^53^, a PPARγ activator (*EDF1*) and a cyclin gene (*CCNP*). Genes upregulated in the torpor + radiation group and downregulated in the radiation group were a kinesin (*KIF25*), and a pyruvate dehydrogenase phosphatases subunit (*PDP1*), (Supplementary Table S16).

A comparison of liver genes with those of the GIT (Supplementary Table S17) showed a shared upregulation of the circadian rhythm pathway (M95), amino acid metabolism (M39570), signs of both cell cycle arrest (GO: 0033048, GO: 0051784) and cell division (GO: 0051301). Analysis of the shared downregulated genes indicates a shared decrease in TCA cycle activity.

### Temperature and melatonin meta-analysis

To dissect the transcriptomic effects of temperature and melatonin we performed a systems-level meta-analysis. First, a gene-wise comparison was made with the DEGs of the temperature + radiation group (Supplementary Table S18)(*q* ≤ 0.4), with the radiation group (*q* ≤ 0.1)(18.5-rad vs 28.5-rad) to find temperature-mediated effects. The comparison found 390 DEGs unique to the temperature + radiation group taken to reflect the effects of temperature (Supplementary Table S19). The ORA of the 215 upregulated DEGs in this group indicates that the temperature was leading to the changes observed in mitosis and the cell cycle (GO: 0000280, GO: 1903047) (Table S18). Downregulated genes suggest temperature dependent changes in the TCA cycle (1,270,121), mitochondrial-related genes (GO: 0098798, GO: 0005743), and translational GO terms (M14691) as expected (Table S20).

We next sought to dissect the effects of melatonin from the model through a gene-wise comparison, performed with the DEGs in the temperature + melatonin-radiation group and the temperature + radiation group (18.5-mel-rad vs 18.5-rad) (q ≤ 0.4), focusing on those that were found to be unique to the 18.5-mel-rad group after comparison (Supplementary Table S21). ORA results from the 161 DEGs unique to the temperature + melatonin-radiation group were further filtered by comparisons with ORA results from the radiation group and those defined as temperature-driven changes (temperature + radiation vs radiation, Table S20) (Supplementary Table S22). Thus, it’s considered that melatonin is leading to increased acetyl-CoA metabolism (GO: 0006637), nucleoside monophosphate metabolism (GO: 0009162), axon regeneration (GO: 0031103), and spindle organisation (GO: 0051294). Downregulated genes were involved in DNA damage checkpoints (GO: 2000003), midbrain morphogenesis (GO: 1904693), the negative regulation of T cell proliferation/morphogenesis (GO: 0045620), and tight junctions (GO: 0061689).

### Phenotypic end point analysis

**Figure 4:**
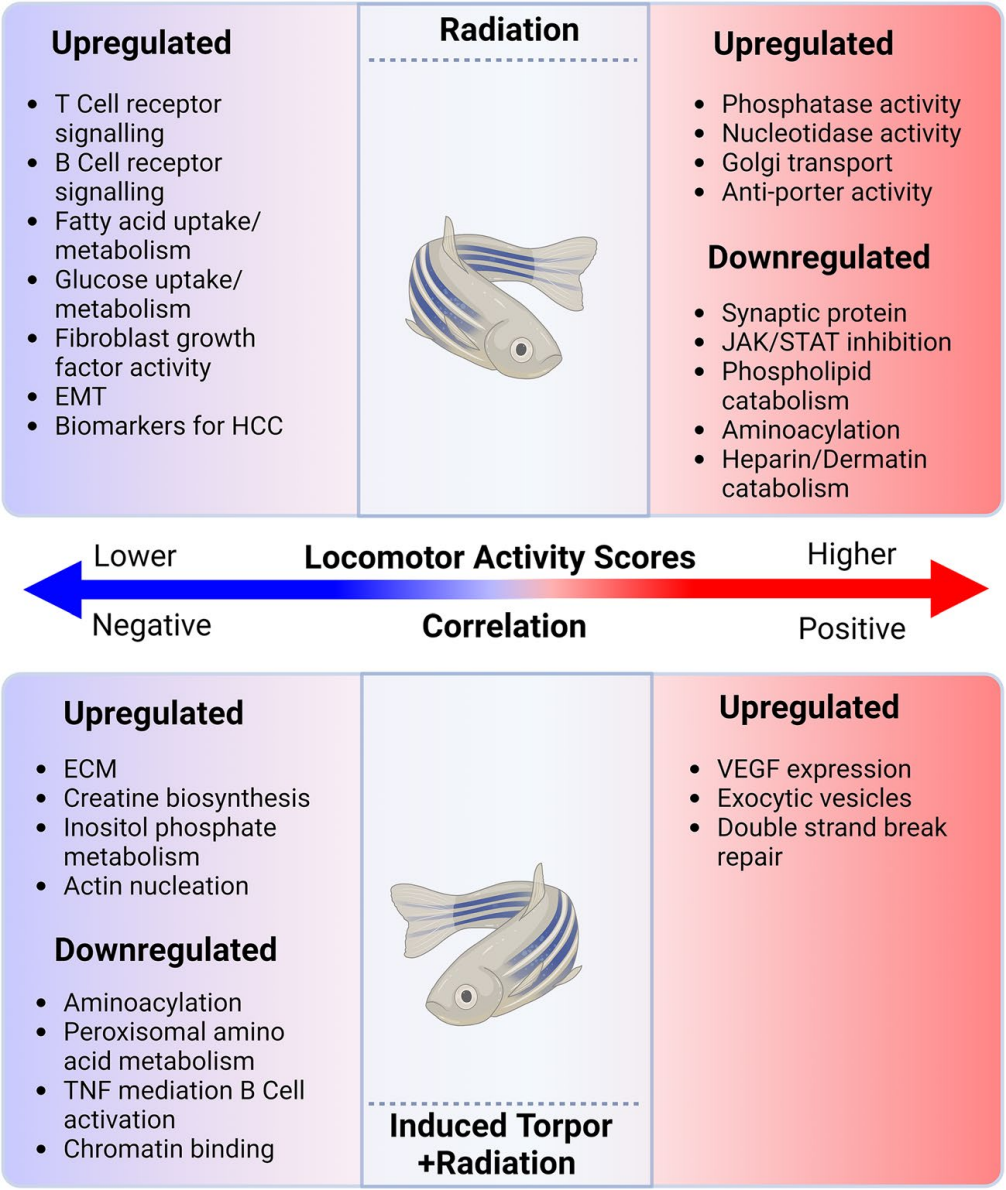
Genes correlated with locomotor activity scores in the radiation and torpor + radiation groups. This schematic shows that increased activity scores in the radiation group are correlated to an upregulation of genes involved in biological processes such as nucelotidase activity, Golgi transport and phagocytosis, and a downregulation of growth, endocytosis, aminoacylation and heparin catabolism related genes. Genes correlated with lower activity scores in the group are associated with immune-related processes such as hemopoiesis, mononuclear cell differentiation, and B cells receptor signalling. In the torpor + radiation group genes correlated with higher activity scores are associated with VEGF expression, exocytic vesicles, and double strand break repair. Genes correlated with decreased activity are correlated with ECM, actin nucleation, creatine biosynthesis and inositol phosphate and a downregulation of genes involved in aminoacylation, amino acid metabolism, B cell receptor activation and chromatin binding. Created with BioRender.com.

Behavioural studies of zebrafish acclimated at 18 °C have previously found a decrease in locomotion^54^. This was supported in our previous work showing that acclimation at 18 °C with the addition of melatonin also led to reductions in locomotion as determined by activity scores in comparison to control groups^37^. These activity scores were generated by tracking the active swimming time, velocity, and time spent by the zebrafish at the bottom or the upper part of the beaker where higher activity scores represent a more balanced use of space, with more frequent movement between the bottom and upper part of the beaker, whereas lower activity scores indicate less movement and more time sent at the bottom of the beaker. We performed a phenotypic end-point analysis by determining the correlation of activity score and DEGs in both the radiation and torpor + radiation groups. First, normality tests of the rlogged DEGs in the radiation group revealed 650 normally distributed genes and 38 not normally distributed genes (Supplementary Table S23). Normality tests on the torpor + radiation DEGs revealed 437 normally distributed genes and 36 non-normally distributed genes (Supplementary Table S24). QQ plots of these genes can be seen in Figure S6. Spearman’s correlation analysis of the remaining 38 non-normally distributed radiation genes and 36 Torpor + radiation genes revealed no correlation. Pearson’s correlation analysis of normally distributed radiation genes found a correlation of 68 genes with the activity scores, 12 of which showed a positive correlation while 56 were negatively/inversely correlated with activity scores. Eleven genes in the torpor + radiation group were correlated, 3 were positively correlated while the remaining eight were negatively correlated. Gene expression values were reappended to the correlated genes (Supplementary Table S25 & S26) and used for ORA analysis (Supplementary Table S27 & S28), the results of which are summarised in Fig. 4.

In the radiation group, ORA of genes positively correlated with locomotor activity suggests roles in diacylglycerol diphosphate/phosphatidate phosphatase activity (*PLPP5*), nucleotidase activity (*NT5DC1*), Golgi transport (*SLC35E1, GOLM2*), and anti-porter activity (*SLC35E1*). While ORA of the downregulated genes suggests decreased aminoacylation (*GARS1*), a structural synaptic gene (*DLG1*), heparin/dermatan sulfate catabolism (*IDUA*), JAK/STAT inhibition (*SOCS2*), and phospholipid catabolism (*PLBD2*). On the other hand, genes inversely correlated with activity scores point to the upregulation of genes involved in B cell and T cell receptor signalling (*MALT1, PTPN6, NFATC2, MAP4K1, REL),* EMT (*ITGB4*), fibroblast growth factor activity (*KLB*), fatty acid uptake/metabolism (*FABP2, SUGCT, LTA4H*), glucose utilisation (*GNPDA1, NEUROD1, SCGN, SLC2A12, SH2D3A, PIK3C2G*), DNA repair (*BARD1*), and gene biomarkers for hepatocellular carcinoma (HCC) (*HELLS, PIWIL2, TROAP*). Similarly, in the torpor + radiation group, upregulated genes positively correlated with activity suggest increased docking of exocytic vesicles (*EXOC1*)*,* double-strand break repair (*BABAM2*), and downregulation of *VEGF* expression (*MAP3K6*). Genes that were negatively correlated or related to decreased activity indicate upregulation of the ECM (*VWA5B2),* creatine biosynthesis (*GATM),* inositol phosphate metabolism *(INPP1),* and actin nucleation *(CORO1B*), suggesting cytoskeleton remodelling. Downregulated genes were involved in aminoacylation *(IARS1),* peroxisomal amino acid metabolism *(LONP2),* chromatin binding *(CRAMP1)* and TNF-mediated B cell activation *(TNFSF13B)* indicating a muted immune response (Fig. 4).

### Comparison of induced torpor in zebrafish with hibernating grizzly bear reveals shared responses

**Figure 5:**
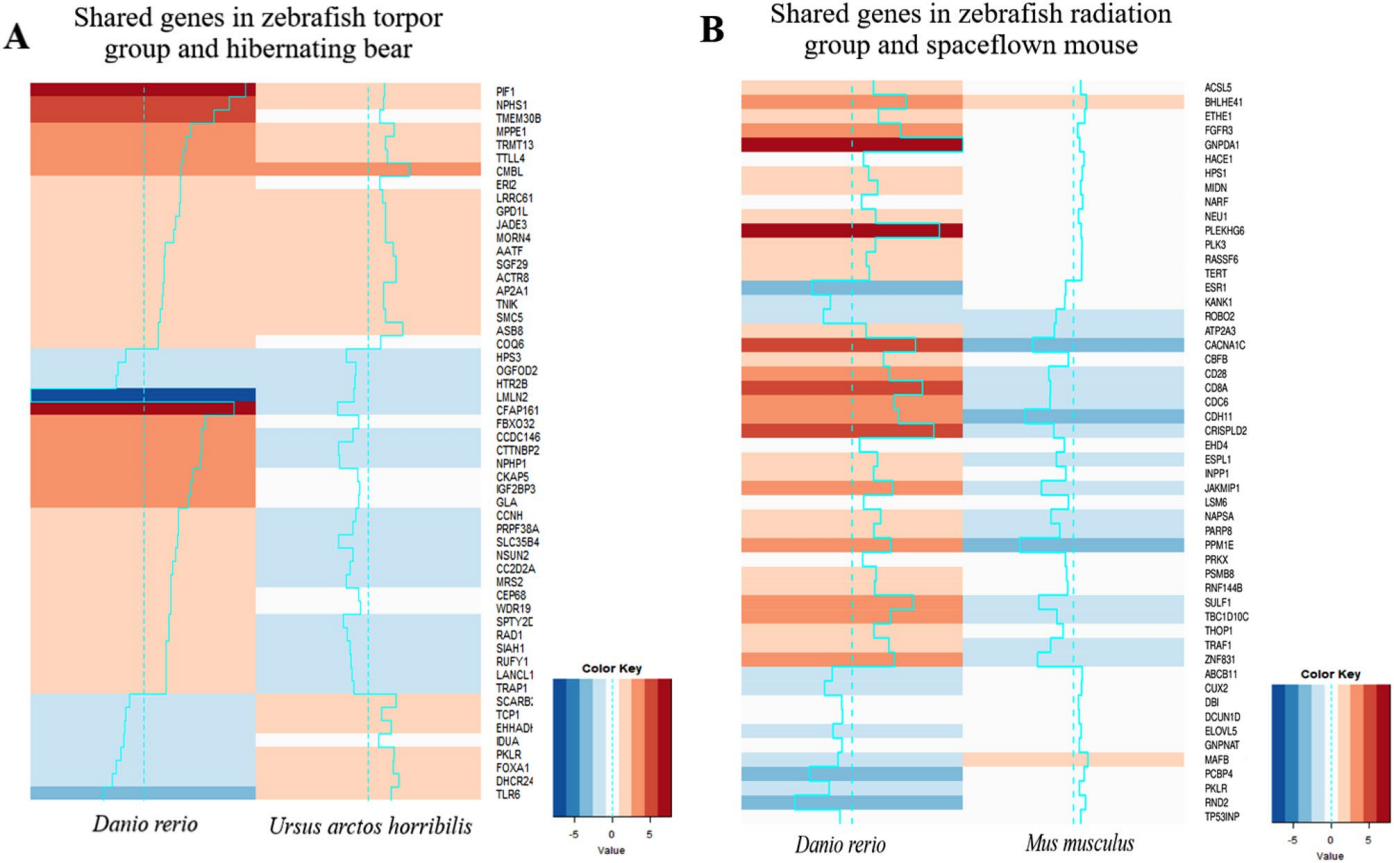
(**A**) Comparison of zebrafish torpor and hibernating bear genes. A heat map of the 54 shared genes between the zebrafish torpor group and the hibernating bear showing their direction of expression relative to the active controls (**B**) Heat map of the 52 shared genes between the zebrafish radiation group and the space flown mouse liver showing their direction of expression (red = upregulated, blue = downregulated).

**Figure 6:**
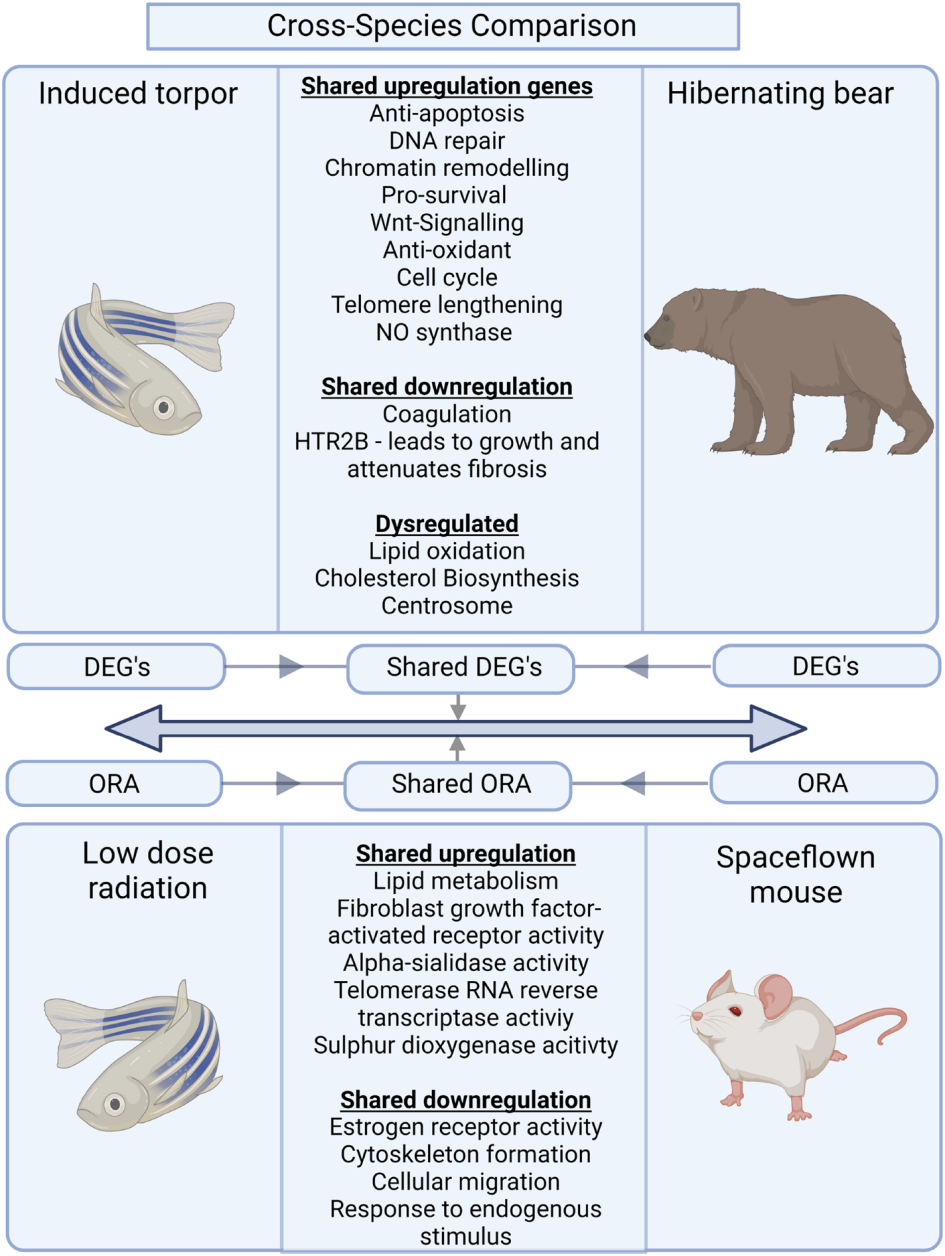
Comparison of our zebrafish radiation group vs space flown mouse and the zebrafish induced torpor group vs hibernating bear. The results between the zebrafish radiation model and the space flown mouse show shared upregulation of genes involved in sulphur dioxygenase activity, telomerase RNA reverse transcriptase, alpha-sialidase activity, and fibroblast growth factor receptor activity. Shared downregulated genes included the estrogen receptor (*ESR1*), and genes involved in cytoskeleton formation and cellular migration. Additionally, shared biological processes included lipid metabolism and response to endogenous stimuli. Comparison of the torpor group versus the hibernating bear showed shared upregulation of anti-apoptotic genes, DNA repair genes, pro-survival genes, antioxidant genes, and Wnt signaling. Shared downregulated genes included anticoagulant genes, while dysregulated genes were involved in lipid oxidation and cholesterol synthesis. Furthermore, shared BP included a response to ROS, cell cycle checkpoints, telomere lengthening, and nitric oxide synthase. Created with BioRender.com.

We sought to explore any shared responses between our hypometabolic model in the zebrafish with that seen during the natural hypometabolic state during torpor of a hibernating animal. Thus, a cross-comparison was performed with DEGs from the zebrafish-induced torpor group (vs control) and those generated from hibernating grizzly bear liver vs control, (Supplementary Table S29). We identified 54 shared genes (*q* ≤ 0.1) shown in Fig. 5A (Supplementary Table S30) and summarised in Fig. 6. Twenty of these genes were upregulated, four were downregulated, and the rest were dysregulated between the groups.

We noted the shared upregulation of *AATF,* which is involved in inhibiting p53-mediated induction of apoptotic genes after DNA damage suggesting shared anti-apoptotic pathways^55^. DNA repair genes were also shared, including *ACTR8* which plays a role in ssDNA synthesis during double-strand breaks during homologous recombination^56,57^. Similarly, a structural maintenance complex gene, *SMC5,* with roles in homologous recombination, was also upregulated^58^ as well as *PIF1*, a DNA synthesis stimulator during break-induced replication^59^. Other genes upregulated in both datasets were involved in chromatin remodelling including (*SGF29)*, which has been implicated in promoting cell survival under ER stress^60^, as well as, *JADE3*, with a role in histone acetylation found to be induced by Wnt/β-catenin signalling^61^. Interestingly, we also found shared upregulation of *TNIK*, involved in the activation of Wnt-signalling and known to promote proliferation or differentiation^62^. We also report upregulation of *COQ6* in both datasets, involved in the biosynthesis of coenzyme Q10, a potent antioxidant shown in zebrafish to protect against ROS-induced apoptosis^63^. Genes downregulated in both species included *HPS3*, a gene involved in dense granule formation with a role in coagulation that could help prevent blood clotting at reduced temperatures^63^. Similarly, *HTR2B* was downregulated: its experimental antagonism has been shown to attenuate fibrosis during liver injury^64^.

A comparison of the shared biological processes (shown in Supplementary Table S31, *p* < 0.05) suggested the shared negative regulation of ROS generation (GO: 1903427) and positive regulation of nitric oxide synthase (GO: 0051000). Cell cycle processes were also returned showing shared meiotic cell cycle checkpoint (GO: 0033313), protein localization to kinetochore (GO: 0034501), and processes related to telomere lengthening (GO: 0032210, GO: 1904356, GO: 0010833). We also observed an over-representation of processes linked to cell projection assembly (GO: 0030031, GO: 0120031, GO: 0060271) and calcitonin family receptor signalling pathway (GO: 0097646).

### Low dose radiation in zebrafish compared with space flown mouse liver

Finally, we wanted to compare the response to radiation in the zebrafish with that of space flown mouse liver using the NASA Genelab database (GLDS-47). The space flown mouse data had 220 DEGs (*q* ≤ 0.4) (Supplementary Table S32), again examined using ORA (*q* ≤ 0.4, Supplementary Table S33). A cross-comparison was used to find shared genes (Supplementary Table S34, Fig. 5B), which were also subject to ORA (Table Supplementary S35). It revealed 52 shared orthologues and the shared upregulation of genes involved in fibroblast growth factor-activated receptor activity *(FGFR3)* (GO:0005007), suggesting radiation-induced fibrogenesis, and *NEU1* involved in cell migration. The mice also showed the upregulation of sulphur dioxygenase activity (*ETHE1*) (GO:0050313), consistent with spaceflight-induced changes to sulphur metabolism^65^. On the contrary, shared downregulated genes include *ROBO2,* which is involved in hepatic stellate cell activation, and *KANK1,* which is involved in cytoskeleton formation. A comparison of the upregulated ORA results found shared GO terms involved in cellular lipid catabolic processes (GO:0044242), while shared downregulated results involved a response to endogenous stimulus (GO:0071495), as summarised in Fig. 6.

## Discussion

### Melatonin as a modulator of lipid metabolism

The liver plays a central role in lipid homeostasis in regulating the uptake, metabolism, redistribution, and excretion of fatty acids. For instance, upon ingestion and absorption from the lumen of the small intestine, dietary fatty acids that are not assimilated into adipose or muscle tissue are re-esterified to triglyceride and taken up by the liver from chylomicrons in the plasma. These can then be stored in intracellular lipid droplets, transferred to peripheral tissue via VLDL (very low-density lipid) particles, or processed back to fatty acids for energy release through β-oxidation in the mitochondria of hepatocytes. Additionally, fatty acids can be generated from glucose via de novo lipogenesis^66^. However, dysregulation of lipid metabolism is associated with insulin resistance^67^, diabetes^67^, and NAFLD, a disease encompassing lipid accumulation, nonalcoholic steatohepatitis, fibrosis, and cirrhosis^68^. Dysregulation of lipid homeostasis has been a well-documented response to the spaceflight environment, with studies showing lipid droplet accumulation^28^, and transcriptional changes in genes involved in lipid localization/transport and lipid/fatty acid metabolism^32^. Further studies show elevated levels of ALT and AST^69^, which indicate liver damage and are often used to aid diagnosis of NAFLD.

Our results from low dose radiation exposure in the zebrafish suggest that exposure led to increased lipid metabolism, transport, and accumulation with increased expression of genes such as *LIPH, ABCA2,* and *FABP2* in the liver. Similarly, we observed an elevated expression of *SCARB1*, which mediates cholesterol efflux. The enrichment of fibroblast growth factor might indicate a fibrotic phenotype. Hence, the results highlight the potential for LDR in increasing the risk of NAFLD-associated symptoms, representing a significant stumbling block to maintaining hepatic health in astronauts. Lipids are the preferred energy source in fat-storing hibernating animals that undergo periods of hyperphagia before entering torpor when triglycerides are then mobilised from fat stores to the bloodstream for energy. This process leads to seasonal hyperlipidemia and hypercholesterolemia, which in humans are risk factors for atherosclerosis or thrombosis. Observations suggest that torpor may be protective against the adverse effects of lipid dysregulation, given that these periods of altered lipid homeostasis do not lead to adverse outcomes. For example, hibernating brown bears undertake long bouts of torpor that leads to limited potential for excreting excess cholesterol. However, they employ mechanisms for advoiding the adverse effects of lipid dysregulation including increased levels of a cardio-protective HDL (*HDL2b*), a re-esterification process to recycle cholesterol, and increased anti-oxidant capabilities to negate oxidative damage from peroxidation^70^. Although, it is unclear if the dysregulation of lipids during spaceflight would interfere with lipid utilisation during torpor, or whether lipid utilisation during torpor would compound the adverse effects of spaceflight. This brings into question the suitability of lipid as the main energy source for space travel. In addition, the feasibility of astronauts gaining the amount of weight that would be needed to sustain a torpor-facilitated trip to Mars has also been called into question, and thus alternative energy sources need to be considered. Melatonin has modulatory effects on lipid metabolism, with studies showing that melatonin treatment reduced serum cholesterol, hypertriglyceridemia, hyperinsulinemia, liver lipid content, and has shown utility in the treatment of diabetes and NAFLD^71^. So its use might prove beneficial in ameliorating the adverse effects of spaceflight. Our data points to a temperature-driven downregulation of metabolic pathways, including pyruvate metabolism and the TCA cycle. However, when combined with radiation, we see the characteristic upregulation of lipid transport and metabolism. *Conversely,* when reduced temperatures are combined with melatonin treatment, we observe a downregulation of lipid metabolic genes, as seen when these processes were dysregulated in the cross-species comparison in Fig. 6. When radiation is added, only lipid biosynthesis is upregulated. While the results suggest that melatonin treatment under hypometabolic conditions might reduce radiation-associated lipid dysregulation by regulating lipid metabolism we recognise that further work is needed to characterise and establish the viability of alternative modes of metabolism under hypometabolic states.

### Hypo-metabolic state confers an anti-apoptotic phenotype

The suppression of apoptosis and increase in pro-survival signalling are key characteristics of the phenotype observed in the hypometabolic groups of the zebrafish GIT and liver. Furthermore, the shared upregulation of *AATF* with the hibernating bear suggests a conserved anti-apoptotic mechanism. For instance, the protein product of *AATF* forms a complex with p53 and BRCA1 to activate cell cycle arrest genes, leading to a cell state favouring survival over apoptosis and allowing more time for repair^72^. This anti-apoptotic phenotype has also been observed in hibernating squirrels with studies reporting an upregulation of anti-apoptotic genes during torpor (*Bcl-2, Bcl-xL, Mcl-1,* and *BI-1*)^73^. A further survival advantage might be conferred through polyamine generation, which was indicated in the induced torpor and torpor + radiation groups through the upregulation of *AMD1* and *OCD1.* These genes promote polyamine biosynthesis, decreased levels of which are associated with increased apoptosis^74^. To continue, the liver and GIT also shared enrichment of the FOXM1 transcription factor network. This has been shown to positively regulate the anti-apoptotic gene *Bcl-2*^75–77^ with additional *FOXM1-*knockdown studies demonstrating its efficacy in promoting survival in human cells after irradiation^78^. Interestingly, *FOXM1* is upregulated in a *HIF-1* dependent manner^79–81^ and the induction of the hypoxia response is known to increase expression of pro-survival genes^82^, suppress apoptosis^83^, and increase proliferation^84^ which are some of the key phenotypes represented in the results. Although our cross-species comparison did not return shared upregulation of *HIF-1*, increased *HIF-1* has previously been reported in the liver of bears, squirrels, and bats during hibernation, implying a widely conserved response in hibernation^85,86^. It may also be plausible to suggest that cold-tolerant species benefit from the upregulation of the hypoxia pathways in response to reduced oxygen, which is achieved and maintained by the reduced metabolic rate to prevent oxygen starvation.

### DNA repair in the induced torpor model of zebrafish

In this paper, we report that the induced torpor model in zebrafish saw an increase in DNA repair processes, including DNA mismatch repair, nucleotide base excision repair, and homologous recombination. This included the upregulation of a sirtuin gene (*SIRT6*), which has a role in DNA repair and promoting longevity^87,88^. Reduced temperatures may have led to an increase in oxidative stress that, in turn, has led to DNA damage. Consistently, results for the torpor + radiation group also showed an upregulation of DNA repair processes. However, given that irradiation occurred 2 days after the initiation of the torpor protocol, prior temperature-induced DNA processes may have preconditioned the cells to radiation stress. This radio-protective preconditioning effect has been evidenced previously in human cells by Lisowka et al., who observed a hypothermia-activated DNA damage response (DDR) that remedied later insult by reducing the number of chromosomal aberrations after irradiation^89^. Temperature-related changes in DNA repair processes have also been shown to be effective in remedying ischemic stress in human clinical trials. For instance, a randomised trial sought to determine if therapeutic hypothermia could treat hypoxic-ischemic encephalopathy in infants: a condition characterised by hypoxic stress that leads to chromosomal aberrations, the number of which correlate with disease severity. The results revealed that reduction of the neonates' body temperatures to 33–34 °C for 72 h following perinatal asphyxia resulted in a statistically significant reduction in the number of chromosomal aberrations compared to the control group, likely through the upregulation of DNA repair genes^90^. This is supported by studies in mice demonstrating that mice preconditioned with sub-lethal ischemia before more severe ischemia can increase ischemic tolerance through induction of DNA repair mechanisms^91^. To continue, when observing hibernating animals, transcriptomic studies of the liver in hibernating 13-lined ground squirrels (*Ictidomys tridecemlineatus*) also found enrichment of genes involved in double-strand break repair with upregulation of genes such as *DDX11* that were correlated with colder body temperatures^92^. Similar observations were made by Jenson et al., who reported DNA repair-related GO terms in the liver of hibernating grizzly bears (*Ursus arctos horribilis*) involving serval DNA repair (e.g. *BABAM2, BRCA2, DCLRE1A*). Although they were not enriched in the adipose or muscle tissue suggesting a tissues-specific response^6^. When comparing hepatic responses in the zebrafish torpor group with results of the hibernating bear, however, we discovered a shared upregulation of DNA repair genes involved in homologous recombination, which might suggest a conserved response to hypothermia in promoting cold resistance.

### Utilisation of partial EMT during torpor may offer radio-protective effects

Epithelial cells are differentiated, polarised cell types attached to a basal membrane by hemidesmosomes and are held together at tight junctions, adherens junctions and desmosomes. During EMT cells lose polarity with degradation of the connections to basal membrane and cell–cell junctions and reorganisation of the ECM, ultimately producing cells with stem-like qualities^93^. There are three types of EMT, which play important roles in development (type I), tissue regeneration (type II), and for malignant growth and metastasis (type III)^94^. Type II EMT is stimulated post-injury to generate new tissue, involving activation of an immune response that produces inflammatory signalling molecules, such as secretion of TGF-β. TGF-β activates mesenchymal cells, such as HSCs and fibroblast cells to produce ECM proteins such as collagens to form fibrotic tissue which acts as a scaffold to guide cell migration^95^. It can also initiate EMT of hepatocytes to acquire a mesenchymal phenotype^96^. When wound healing is complete, inflammatory signals subside but chronic insult can lead to accumulation of fibrotic tissue that impacts liver structure and can lead to liver dysfunction associated with the development of NAFLD, cirrhosis, or cancer^97,98^. Our results for zebrafish exposed to LDR show upregulation of genes involved in EMT, an immune response, and genes involved in fibroblast activity. This might suggest immune-mediated activation of a fibrotic type II EMT programme in a wound healing response to radiation-induced damage. Consistently, Wang, S. et al*.* studied the response to 6 Gy radiation in mice liver at 6–10 weeks post-irradiation finding an increase in fibrosis at 10 weeks, post-irradiation. They found an increase in liver progenitor cells at 6 & 10 weeks, and an increase in expression of pro-fibrogenic cytokine (TGF-β) at 6 weeks, along with an increased markers for activated HSC’s (α-SMA) and EMT (s100a4, N-cadherin, and collagen α1) at 6 & 10 weeks post-irradiation. Moreover, they show a role for the hedgehog signally pathway in generating the fibrotic phenotype^27^. Jonscher et al., reported a similar trend, revealing that mice exposed to 2 weeks of spaceflight experienced profibrogenic markers in the liver involving an increase in *Lepr*, which can promote myofibroblastic HSC transition, as well as, *PAI-1* and TGF-β receptor subunits (Tgfbr1, 2 & 3)^28^. Additionally, Tian et al. found increased profibrotic lesions in air spaces and blood vessels in the lung of 13-day spaceflight-exposed mice^99^.

Interestingly, a partial/reversible EMT has been observed in lung tissue of hibernating animals, evidenced in a study of Syrian hamster (*Mesocricetus auratus*) lungs that followed EMT markers throughout different stages of hibernation. They found high expression levels of *TGF-β*, collagen, and smooth muscle actin during early torpor which returned to normal after torpor^100^. Similarly, a study on the brains of thirteen-lined ground squirrels observed decreased epithelial markers (E-Cadherin) and an increase in EMT markers such as vimentin and *Sox2* which were restored to pre-entry expression levels at the arousal stage. The expression of miRNA families (mir-200 & mir-182) known to control EMT was also correlated with the expression of EMT markers, further supporting the reversible regulation of EMT between the torpor and active states^78^. In concurrence, DEA of the hibernating bear compared with active controls revealed upregulation of ECM-related genes such as collagen (*COL4A4, COL17A1*) which have been shown to promote EMT in response to TGF-β^101^. Similarly, the downregulation of laminin (*LAMB1*), which forms a complex with integrins to promote cell adhesion^102^, may facilitate EMT. To continue, the torpor group also displayed markers of EMT with the upregulation of collagen genes (*COL4A4, COL17A1*) and matrix metalloproteinase (*MMP17*) as well as the downregulation of protocadherin genes (*PCDH12, PCDH17*) and plakophilin 2 (*PKP2*). We know that the immune system plays an important role in fibrotic development^103^, however, immune activity is regularly reported to be downregulated during hibernation^45^. Hence, immune regulation during torpor might be protective against a fibrotic phenotype. In support of this view, a striking observation made by Andres–Mateos et al., in a study of skeletal muscle in thirteen-lined ground squirrels found that muscle injury did not increase fibrotic tissue levels during hibernation despite the presence of inflammation. They also found that muscle regeneration occurred post-hibernation without the presence of fibrotic tissue^104^. Furthermore, a study that examined the effects of ischemic reperfusion injury in the liver of mice found that hypothermia improved ischemic reperfusion injury and improved hepatocellular function by attenuating an inflammatory immune response^105^. We also observed downregulated immune processes in the induced torpor and torpor + radiation groups, in contrast to the radiation-only group. In addition, we note the shared downregulation of *HTR2B* between the torpor group and the hibernating bear, which has been shown to prevent fibrosis when inhibited^64^. However, further work is needed to determine whether these models evaded fibrotic development. Furthermore, what is interesting is that DNA repair processes and an anti-apoptotic phenotype have also been associated with EMT (reviewed further by Chakraborty et al.^106^). Hence, it may have a role in generating the pattern of gene expression reported here. Additionally, hypoxia has been implicated in promoting EMT^107,108^. Therefore, epithelial plasticity may be exploited in the liver during torpor/hypothermia as a mechanism of imparting resistance to stress, but further work should be conducted to test this hypothesis.

### Radiation exposure may impact zebrafish behaviour

Behavioural analysis previously showed a reduction in locomotion in fish subjected to reduced temperatures with the addition of a sedative (melatonin)^37^. Here, we correlated gene expression data with the behavioural data from the radiation and torpor + radiation groups to draw inferences on gene expression that link to a phenotypic end-point. We note that genes correlated with low activity irradiated zebrafish were involved in a B cell and T cell-mediated immune response that was accompanied by upregulation of genes involved in fibroblast activity and EMT. We also note the correlation of genes involved in the use of glucose and lipids, which might indicate an increase in energy use that is consistent with an energy-expensive stress response. This is in line with previous work, linking increased immune responses in zebrafish with decreased locomotor activity and is indicative of sickness behaviour^50,109^ which in zebrafish presents as lethargy and decreased social or exploratory behaviour^110^. Moreover, work by Cantabella et al. also showed that chronic exposure to low dose radiation reduced locomotion in zebrafish^111^. Interestingly, fish with lower activity scores in the torpor + radiation group show a correlation with downregulated genes involved in B cell activation suggesting a diminished immune response.

### Further work

We found that the induced torpor group appears to increase mitogenic signals. Cells with increased proliferative rates typically become more radio-sensitive due to the vulnerability of DNA to damage during replication. However, it is unclear whether in our model the rate of proliferation is above basal levels or whether increased proliferative signals are needed to maintain cell viability without increasing proliferation against a background of reduced metabolism. Further work might seek to define proliferation rates and mutation rates given the potential for mutagenesis. We also note that epithelial plasticity, including EMT may play a role in torpor-like states and further work is needed to determine how EMT signalling might exacerbate or ameliorate radiation-induced fibrosis in the liver. Moreover, the literature points to hypoxia/cold-tolerant species that use a reduction of metabolism to avoid oxygen starvation and the induction of a hypoxia response. Future efforts could aim to define whether targeting hypoxia pathways can enable a conferred stress resilience. Additional work might also seek to define the relationship between the reduction in metabolism and the acceptable reduction in oxygen content without damage in human cell lines or organoids. Similarly, it would be interesting to define the relationship between this response and the suggested increase in DNA repair capacity, measuring DNA damage relative to gene expression level and level of stressors.

## Conclusion

We investigated the mechanism through which a synthetic/induced torpor protects against radiation exposure using a systems biology approach. We found that induced torpor led to a reduction in metabolic genes and increased pro-survival signalling including proliferative and anti-apoptotic biomarkers that may influence cell fate decisions to avoid cell death. We also observed an increase in DNA repair genes. An increase in DNA repair mechanisms before the stress exposure may act as a buffer to further insult through pre-conditioning. Additionally, we observed that these protective mechanisms are present under induced torpor with radiation, suggesting maintenance of these mechanisms during periods of stress. Furthermore, we note markers of EMT in both the hypometabolic and the radiation group. It was found that the EMT profile in the radiation group was accompanied by immune-related GO terms that could mediate a more fibrotic response. On the other hand, it is possible that an EMT- like programme that does not involve immune regulation imparts stem-like traits that may have a role in stress resilience. Further work will explore the prospect of exploiting induced torpor as a radio-protectant, representing a potential therapeutic strategy to mitigate cellular damage during space travel and in clinical medicine.

## Methods

### Zebrafish husbandry

We obtained strain AB *Danio rerio* (zebrafish) from the Zebrafish International Resource Centre. Adult zebrafish were housed at a maximum density of 6 fish per 1 L glass beaker in an incubator at 28.5 °C with a light cycle of 14 h ON (light) and 10 h OFF (dark). They were maintained and crossed following standard housing methods. Lids were used to prevent fish mortality from jumping out of the beaker while also allowing air flow. Fish were fed Gemma Micro 300 standard diet every other day (Skretting, Westbrook, ME, USA) in the morning and remaining debris was aspirated from the beaker 20 min after feeding. Moreover, to increase water life support capability, 75% of the water was changed daily using reservoir water (Reverse Osmosis water supplemented with Instant Ocean salts, sodium bicarbonate and Stress Coat, maintained at pH 7.4). The beakers used to house fish were cleaned and autoclaved before use. All procedures were performed per The Medical University of South Carolina (MUSC), Institutional Animal Care and Use Committee (IACUC) guidelines (IACUC-2018-00278). All animals were treated with regard for alleviation of suffering. In addition, these procedures followed the ARRIVE guidelines^112^.

### Development of the induced torpor model and radiation protocol

Several experimental groups were used to assess the value of induced torpor as a countermeasure to radiation, detailed in Table 1. First, a control group was maintained at an ambient temperature of 28.5 °C (28.5-Ctrl). Second, a melatonin group (28.5-mel) was established that received 24 µM of melatonin daily (10 days) to reduce locomotion and arousal. When replacing 75% of water daily, we added a mixture of saline water and melatonin to maintain levels of melatonin, protecting against loss due to metabolism and degradation^113^. Melatonin was purchased from Sigma-Aldrich (St. Louis, MI, USA) with ≥ 98% purity, maintained at − 20 °C in powder and dissolved in DMSO before use. Then, a reduced temperature group (18.5-Ctrl) was acclimatised to 18.5 °C to decrease their metabolism. Ambient temperatures were reduced in weekly decrements of 2.5 °C, over 4 weeks to avoid thermal shock^114^. Next, the induced torpor group (18.5-mel) was acclimatised to 18.5 °C and administered 24 µM of melatonin. Again, after 4-week acclimatisation, melatonin was added for 10 days. Subsequently, we established a low dose radiation group (28.5-rad), exposing them to a total whole-body dose of 32.68 cGy. The zebrafish were anaesthetised with 0.02% tricaine prior to radiation exposure and placed one at a time in 60 mm × 15 mm Petri dishes in water on top of a 3-inch spacer ready for irradiation. Radiation exposure occurred for 6 s at 163.40 cGy/min resulting in total exposure of 16.34 cGy, which occurred on both the 2nd and 8th day of the experimental timeline. Irradiation was carried out at MUSC in accordance with IACUC-2018-00278, using the Shepherd Model 143–68, Serial Number 8020 irradiator (JL Shepherd and Associates, San Fernando, CA, USA), with a Caesium 137 radiation source. After radiation exposure, fish were placed in a temporary tank free of tricaine to recover and then transferred back into the main tank with other fish from the same experimental group. An induced torpor group with reduced temperatures and melatonin was also subject to radiation exposure (18.5-mel-rad). The fish were sacrificed on the 10th day of the experimental timeline (starting at the end of the acclimatisation protocol). See Fig. 1 Cahill et al.^37^ for the diagram representing the experimental timeline.

**Table 1:** Experimental groups showing the group names, key, sample number per condition, values of radiation exposure, ambient water temperature, and melatonin treatment.

Group	Key	Sample (N)	Radiation (cGy)	Water temperature (°C)	Melatonin (µM)
Control	28.5-Ctrl	6	0	28.5	0
Melatonin	28.5-mel	6	0	28.5	24
Temperature	18.5-Ctrl	6	0	18.5	0
Temperature + melatonin	18.5-mel	6	0	18.5	24
Radiation	28.5-rad	6	32.64	28.5	0
Temperature + radiation	18.5-rad	6	32.64	18.5	24
Temperature + melatonin + radiation	18.5-mel-rad	6	32.64	18.5	24

### RNA extraction and sequencing

Total mRNA was extracted from liver tissue from the control group (28.5-Ctrl, *n* = 2), radiation group (28.5-rad, *n* = 3), temperature + melatonin (induced torpor) group (18.5-mel, *n* = 2), temperature + radiation group (18.5-rad, *n* = 3) and torpor + radiation group (18.5-mel-rad, *n* = 4) zebrafish using the Qiagen miRNeasy Mini kit (Qiagen, Hilden, Germany). To prepare mRNA-Seq libraries the TruSeq RNA Sample Prep Kit (Illumina, San Diego, CA, USA) was utilised; 100 ng of total input liver RNA was used in accordance with the manufacturer’s protocol. High throughput RNA sequencing (RNAseq) was performed at the Queen’s University Belfast, Genomics Core Technology Unit on an Illumina Next SEQ 500 instrument with the mRNA library sequenced to a minimum depth of 50 million reads using a SE50 strategy.

### RNA-seq data processing and differential expression

FastQC^115^ was used to assess sequence quality, and to identify over-represented sequences and low-quality reads for which Cutadapt was subsequently used^116^. Next, the STAR aligner^117^ was used with align the reads to the zebrafish genome (GRCz11), and subsequently HTSeq^118^ was used to extract read counts per transcript. DESeq2^119^ was utilised to determine the DEG of the experimental groups in comparison with the control group. An identity plot was produced to show sample-sample distances (Supplementary Fig. S8). Thresholds were applied to the DEG using their absolute fold change (linear FC of ± 1.5, or log2FC of ± 0.58) and an FDR adjusted *p*-value (*q* ≤ 0.1), calculated using the Benjamini–Hochberg procedure^120^. Ensembl human orthology^121^ was exploited, appending human orthologs to corresponding zebrafish gene IDs, to leverage the better annotation of human genes, as demonstrated by Huff et al*.*^122^.

### Pathway analysis

Over-representation analysis (ORA) was performed in ToppFunn^123^ with orthologous human gene symbols to define enriched gene ontologies and KEGG pathways^39^ taking either up or downregulated genes as input. The pathway impact analyses were performed using iPathwayGuide (Advaita Bioinformatics, Ann Arbor, MI, USA)^124^ to take advantage of a topology-based approach that considers the type, function, position, and interaction between genes in each pathway to help reduce false positives. Finally, STRING^125^ was exploited to generate protein interaction networks, and g:Profiler^126^ was used in conjunction with EnrichmentMap^127^ to generate GO term clusters using Cytoscape v3.9.1^128^.

### Phenotypic end-point analysis

Phenotypic end-point analysis was performed on the radiation and torpor + radiation groups to determine the correlation between DEGs and activity scores (determined previously in Table S1 of Cahill et al.^37^) in each group. Briefly, GraphPad Prism 8.4.3(San Diego, CA, USA) was used to assess the normality of normalised rlog counts from DEGs with the Shapiro-Wilks test. Correlation analysis was then performed using Pearson’s correlation statistics on normally distributed gene counts against activity scores in each group, while Spearman’s correlation test was performed on those not normally distributed. Expression values were reappended to those genes that were found to be positively or inversely correlated with activity scores (*p* < 0.05) and were gene number permitted. ORA was performed on those genes using Toppfun.

### Model validation

To validate our model of induced torpor and the low dose radiation protocol we compared it with hibernating versus active *Ursus arctos horribilis* (grizzly bear), as well as space flown versus ground control mice. Transcriptomic data from the liver of six active and hibernating bears was obtained from Genbank BioProject PRJNA413091. Similarly, transcriptomic data from the liver of 14 space flown and 14 ground control 16-week-old female C57BL/6 J mice were obtained from NASA’s GeneLab repository (GLDS-47). DEGs were generated for these datasets following the pipeline described in Sect. 2.4. Human orthologs were added to the respective bear or mouse gene IDs using Ensembl orthology and impact analysis was performed in Advaita’s IPathwayGuide, as described above. To characterise the models, a meta-analysis was carried out between the DEGs from the liver of the zebrafish torpor group and the liver of hibernating grizzly bears, as well as on the space-flown mice and the radiation group of the zebrafish.

### Experimental validation

Briefly, a total of 0.1 µg of RNA from 2 biological replicates from the control (28.5-ctrl) and Torpor + radiation (18.5-mel-rad) groups were used in the synthesis of cDNA, using the iScript Reverse Transcription Supermix (Bio-Rad) (4 µl), and Iscript Reverse transcriptase (1 µl) and nuclease-free water to give a total volume of 20 µl. The reaction protocol is described in Supplementary Table S1. Next, two technical replicates of 2.4 ng (2.4 µl) of cDNA were used in the quantification of gene expression performed using SYBR Green qPCR (10 µl) (ThermoFisher, Waltham, MA, USA) on a Roche LightCycler 480 Instrument II (Roche Diagnostics, Rotkreuz, Switzerland). Forward and reverse primers designed using Primer-Blast^129^ (Supplementary Table S2) were added to the reaction (1.6 µl). Primers are not exon junction spanning, however, DNAase treatment was carried out on RNA before cDNA conversion. Then, 6 µl was added to give a total reaction volume of 20 µl. The qPCR thermocycler protocol is described in Supplementary Table S3. The relative induction of gene mRNA expression was then calculated using *actn2b* expression as a reference gene for normalisation, and values for the experimental group (18.5-mel-rad) were compared with values from the control group (28.5-ctrl). T-tests were performed to test for significance and results were plotted using GraphPad Prism 8.4.3 (San Diego, CA, USA).

### Institutional review board statement

This study was carried out in strict accordance with the recommendations in the Guide for the Care and Use of Laboratory Animals of the National Institutes of Health. The procedures described were executed at and with approval of Medical University of South Carolina following the guidelines of the American Association for Laboratory Animal Science (IACUC) under the approved document IACUC-2018-00278.

## Supplementary Information

Supplementary Information 1.

Supplementary Information 2.

Supplementary Information 3.

Supplementary Information 4.

Supplementary Information 5.

Supplementary Information 6.

Supplementary Information 7.

Supplementary Information 8.

Supplementary Information 9.

Supplementary Information 10.

Supplementary Information 11.

Supplementary Information 12.

Supplementary Information 13.

Supplementary Information 14.

Supplementary Information 15.

Supplementary Information 16.

Supplementary Information 17.

Supplementary Information 18.

Supplementary Information 19.

Supplementary Information 20.

Supplementary Information 21.

Supplementary Information 22.

Supplementary Information 23.

Supplementary Information 24.

Supplementary Information 25.

Supplementary Information 26.

Supplementary Information 27.

Supplementary Information 28.

Supplementary Information 29.

Supplementary Information 30.

Supplementary Information 31.

Supplementary Information 32.

Supplementary Information 33.

Supplementary Information 34.

Supplementary Information 35.

Supplementary Information 36.

## Data Availability

The data supporting the findings of this study are openly available in NCBI Gene Expression Omnibus and are accessible through GEO Series accession number GSE200212 at https://www.ncbi.nlm.nih.gov/geo/query/acc.cgi?acc=GSE200212. The following secure token has been created to allow review of the GSE200212 record while it remains in private status: knmfgyksffmtlwv.

## Abbreviations

DEA: Differential expression analysis
DEG: Differentially expressed genes
DRC: DNA repair capacity
DDR: DNA damage response
ECM: Extracellular matrix
EMT: Epithelial mesenchymal transition
ETC: Electron transport chain
FC: Fold change
GIT: Gastrointestinal tract
HSC: Hepatic stellate cells
IACUC: Institutional Animal Care and Use Committee
LDR: Low dose radiation
MUSC: Medical University of South Carolina
NAFLD: Non-alcoholic fatty liver disease
ORA: Over-representation analysis
ROS: Reactive oxygen species
TCA: Tricarboxylic acid
VLDL: Very low-density lipid
