# Supplementary material for: Investigating the effects of chronic low-dose radiation exposure in the liver of a hypothermic zebrafish model
Source: Sci Rep. 2023 Jan 17;13:918. doi: 10.1038/s41598-022-26976-4 (PMC9845366; doi:10.1038/s41598-022-26976-4)
Supplement: Supplementary file 1 — Supplementary Information 1. [file 41598_2022_26976_MOESM1_ESM.docx]

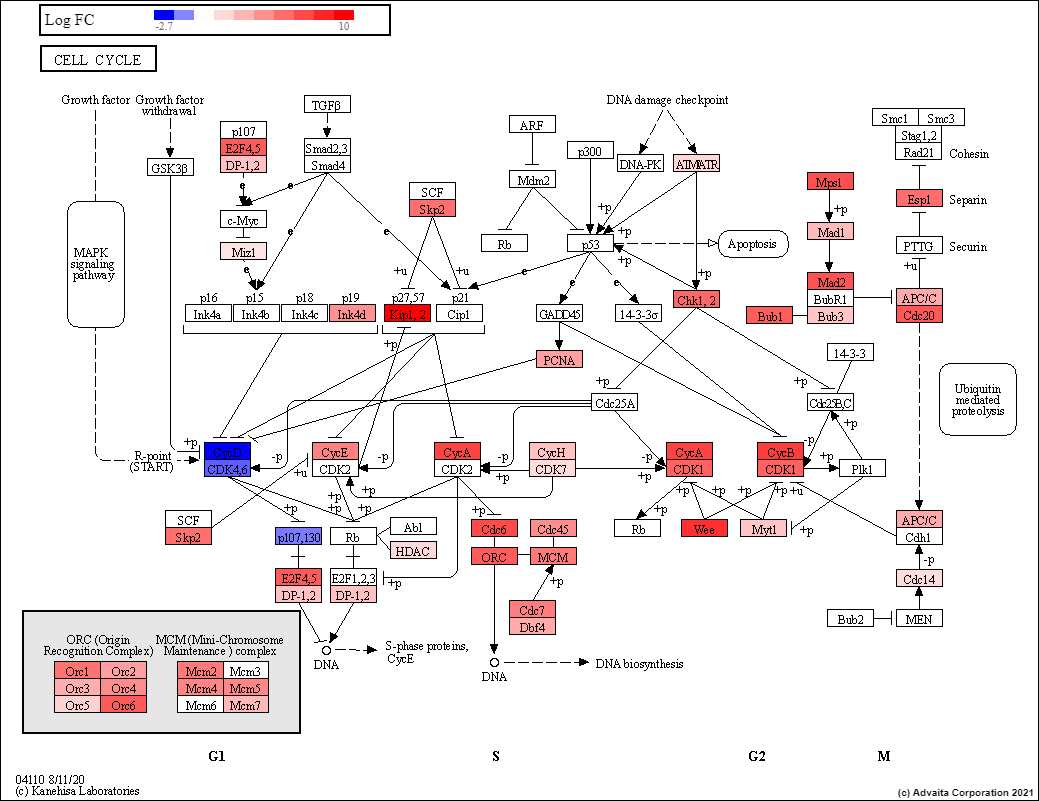
**Supplementary Figure S1: Cell Cycle.** A schematic diagram showing the upregulation of cyclins *CYCA, CYCB* and *CYCE* and cyclin dependent kinases, *CDK1*, in the cell cycle pathway in the torpor group vs control (Image from iPathwayGuide from AdvaitaBio)^1^.

**
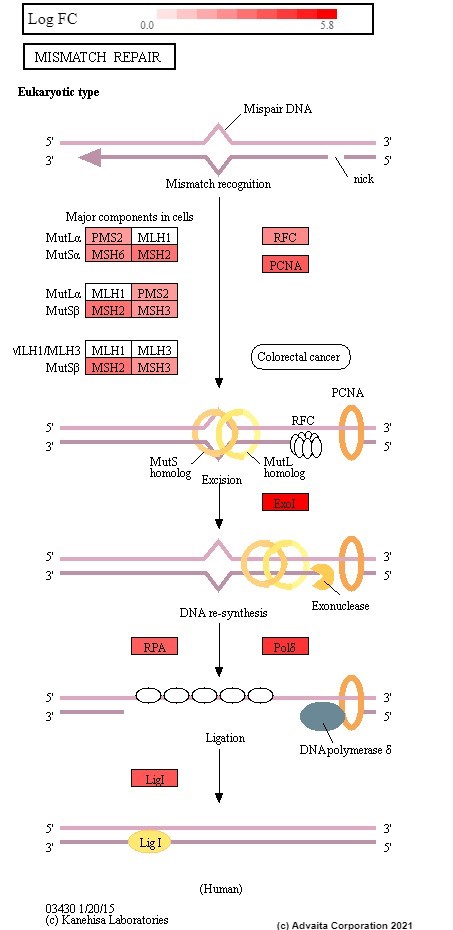
Supplementary Figure S2: Mismatch Repair.** A schematic diagram showing the upregulation of genes involved in mismatch recognition, excision, DNA resynthesis and ligation in the DNA mismatch repair pathway in the torpor group vs control (Image from iPathwayGuide from AdvaitaBio)^1^.

**Supplementary Figure S3: Nucleotide excision repair.** A schematic diagram showing the upregulation of genes involved in the Holo-TFIIH complex in DNA unwinding, incision, excision and ligation in the Nucleotide excision repair pathway in the torpor group vs control (Image from iPathwayGuide from AdvaitaBio)^1^.


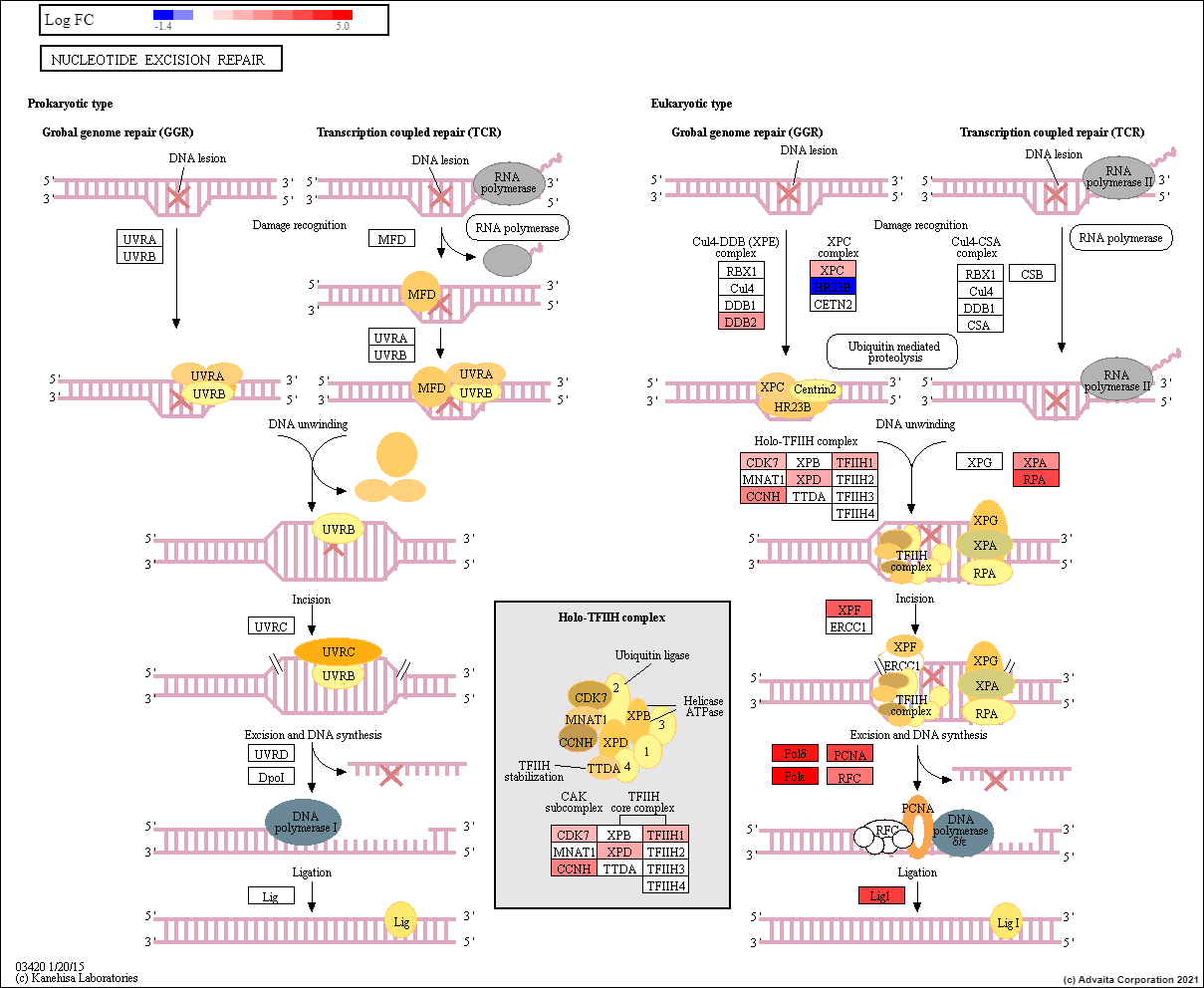


**Supplementary Figure S4: Base excision repair**. A schematic diagram showing the upregulation of genes such as AP-endonuclease and those involved in gap filling and strand displacement in the Base excision repair pathway in the torpor group vs control (Image from iPathwayGuide from AdvaitaBio)^1^.


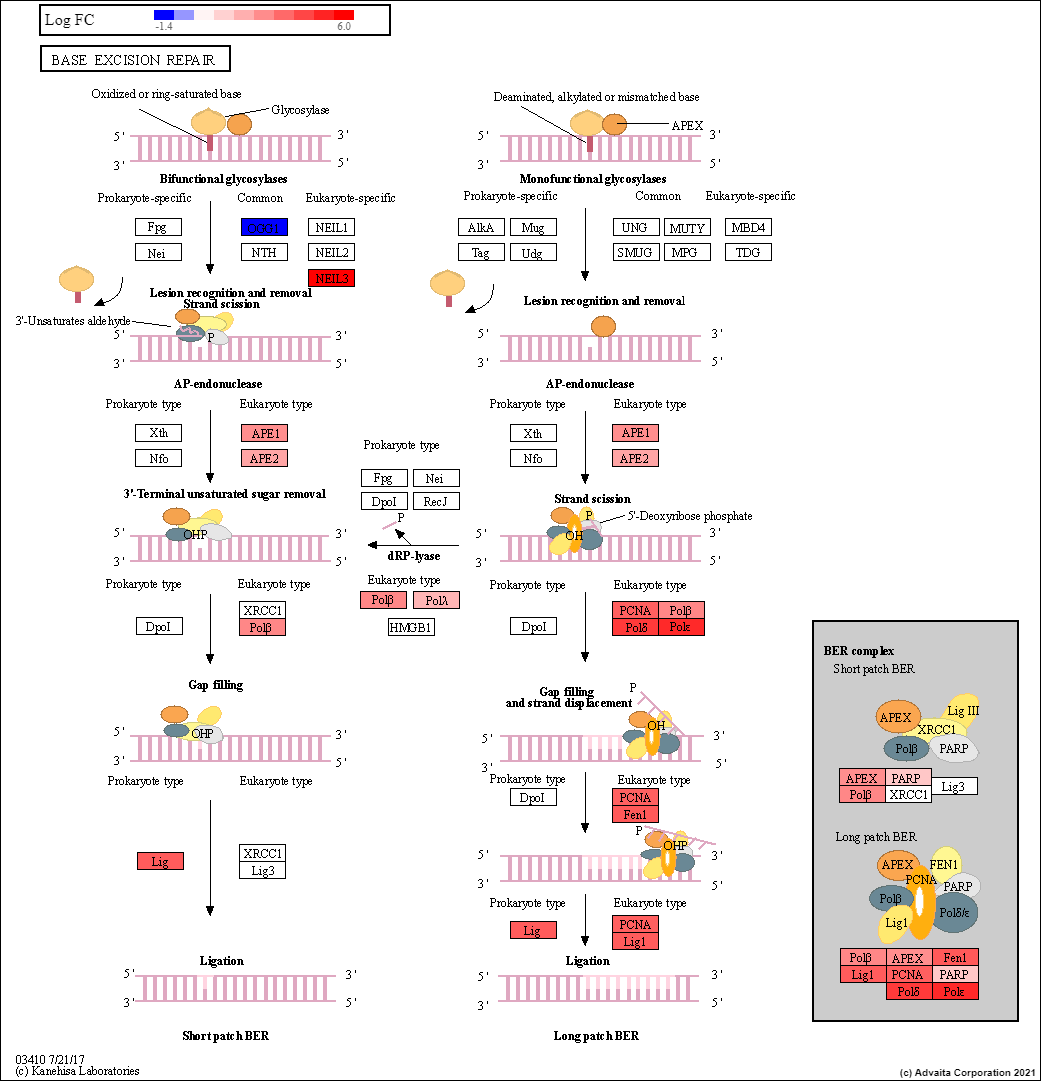


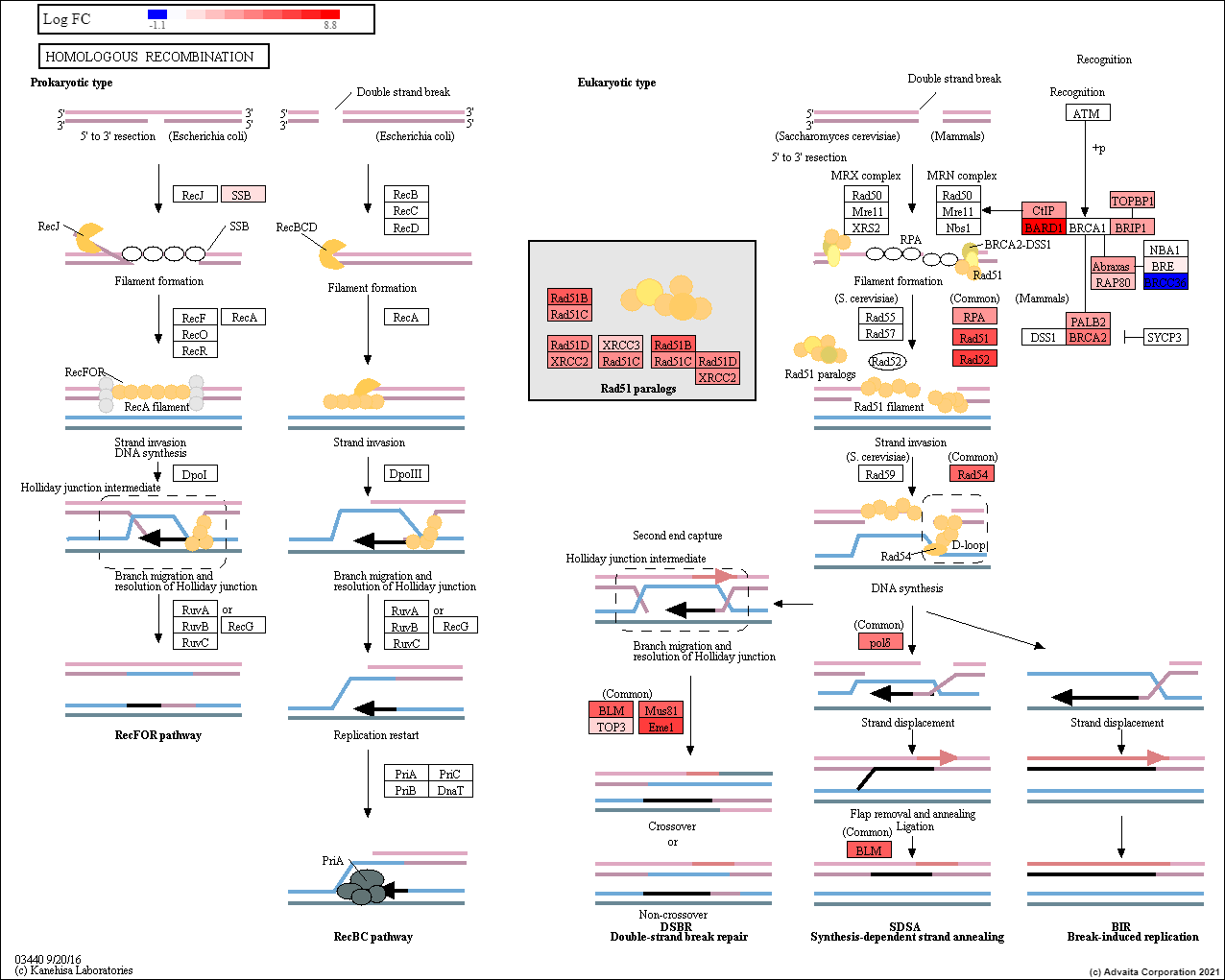
**Supplementary Figure S5: Homologous recombination.** A schematic diagram showing the upregulation of genes involved in filament formation (*RAD51, RAD52*), and strand inversion (*RAD54*) in the Homologous recombination pathway in the torpor group vs control (Image from iPathwayGuide from AdvaitaBio)^1^.

**Supplementary Figure S6: QQ plots.** QQ plots showing the normality of differentially expressed genes in the radiation and torpor+radiation groups supplemented by the number of genes normally and not normally distributed and positively or negatively correlated with activity scores.


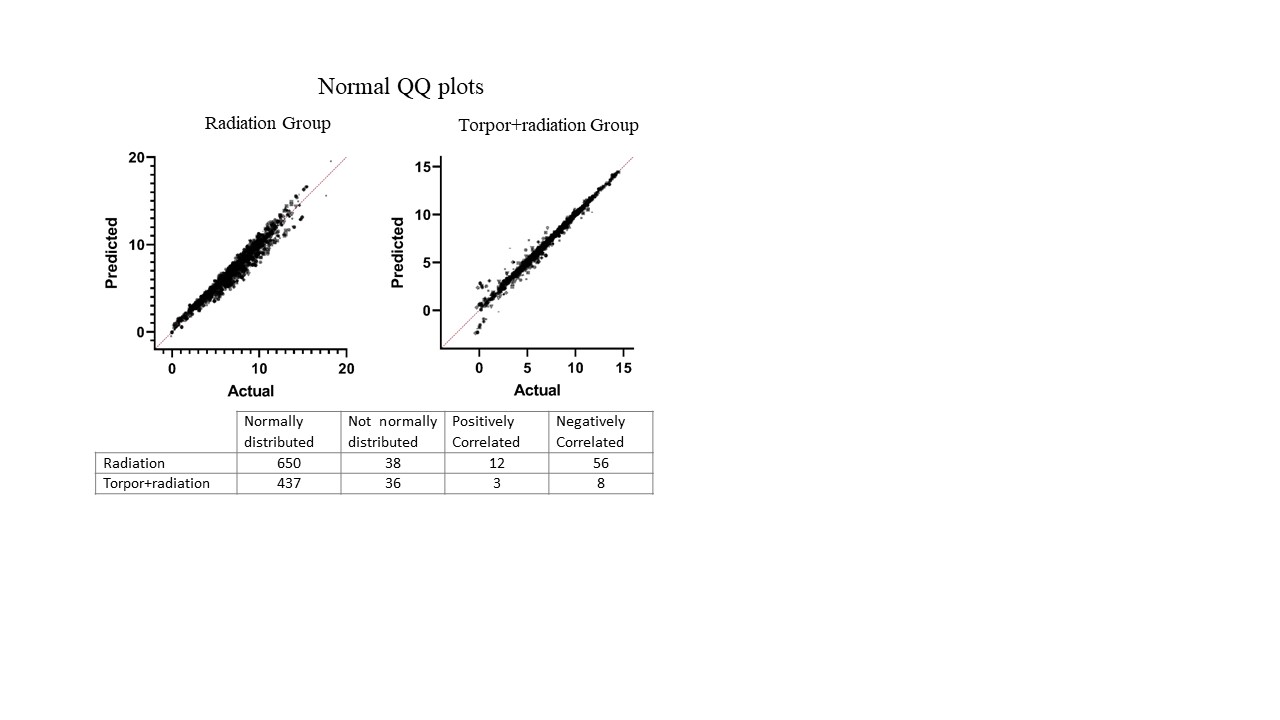


**Supplementary Figure S8. Identity Plot.** Identity plot showing distance matrix of experimental groups and control group displaying similarities and differences between samples.


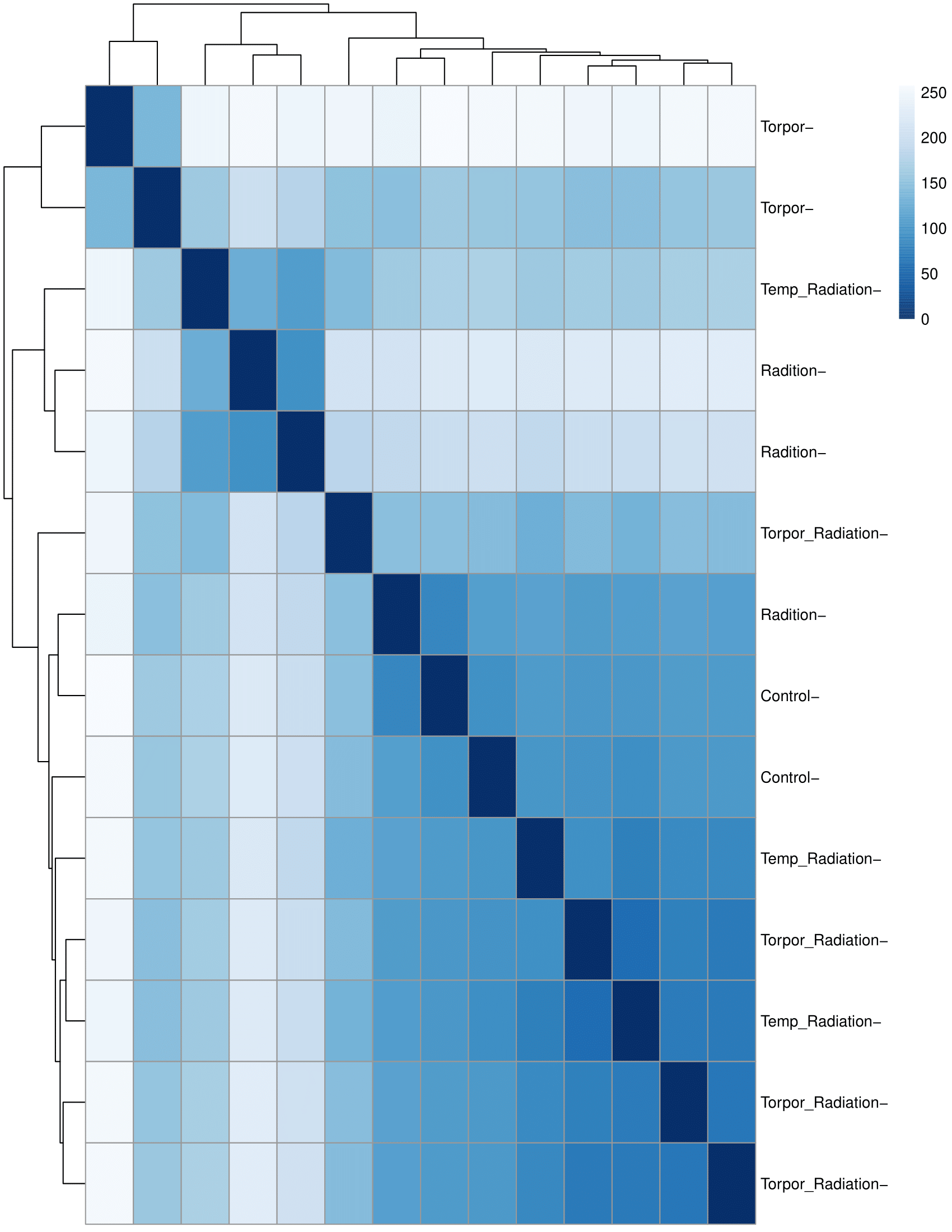


1 Kanehisa, M., Sato, Y., Kawashima, M., Furumichi, M. & Tanabe, M. KEGG as a reference resource for gene and protein annotation. *Nucleic acids research* **44**, D457-D462 (2016).
